# Systematic review on intentional non-medical fentanyl use among people who use drugs

**DOI:** 10.3389/fpsyt.2024.1347678

**Published:** 2024-02-13

**Authors:** Vivian W. L. Tsang, James S.H. Wong, Jean N. Westenberg, Noor H. Ramadhan, Hasti Fadakar, Mohammadali Nikoo, Victor W. Li, Nick Mathew, Pouya Azar, Kerry L. Jang, Reinhard M. Krausz

**Affiliations:** ^1^ Department of Psychiatry, Faculty of Medicine, The University of British Columbia, Vancouver, BC, Canada; ^2^ Complex Pain and Addiction Service, Vancouver General Hospital, Vancouver, BC, Canada; ^3^ BC Mental Health & Substance Use Services, Provincial Health Services Authority, Burnaby, BC, Canada

**Keywords:** addiction, fentanyl, overdose crisis, substance use, opioid use disorder

## Abstract

**Objectives:**

Fentanyl is a highly potent opioid and has, until recently, been considered an unwanted contaminant in the street drug supply among people who use drugs (PWUD). However, it has become a drug of choice for an increasing number of individuals. This systematic review evaluated intentional non-medical fentanyl use among PWUD, specifically by summarizing demographic variance, reasons for use, and resulting patterns of use.

**Methods:**

The search strategy was developed with a combination of free text keywords and MeSH and non-MeSH keywords, and adapted with database-specific filters to Ovid MEDLINE, Embase, Web of Science, and PsychINFO. Studies included were human studies with intentional use of non-medical fentanyl or analogues in individuals older than 13. Only peer-reviewed original articles available in English were included.

**Results:**

The search resulted in 4437 studies after de-duplication, of which 132 were selected for full-text review. Out of 41 papers included, it was found that individuals who use fentanyl intentionally were more likely to be young, male, and White. They were also more likely to have experienced overdoses, and report injection drug use. There is evidence that fentanyl seeking behaviours are motivated by greater potency, delay of withdrawal, lower cost, and greater availability.

**Conclusions:**

Among PWUD, individuals who intentionally use fentanyl have severe substance use patterns, precarious living situations, and extensive overdose history. In response to the increasing number of individuals who use fentanyl, alternative treatment approaches need to be developed for more effective management of withdrawal and opioid use disorder.

**Systematic review registration:**

https://www.crd.york.ac.uk/prospero/, identifier CRD42021272111.

## 1 Introduction

Fentanyl and its analogues such as alfentanil, sufentanil and remifentanil were first introduced into clinical practice (carfentanil in veterinary medicine) as μ-opioid receptor agonists and potent relatively short-acting analgesic agents. Fentanyl is between 50-fold and 100-fold more potent than morphine, thereby offering greater ability to manage intractable pain, breakthrough cancer pain and to produce balanced intravenous (IV) anaesthesia (1). The unique pharmacological properties of fentanyl and its widespread prevalence in the current North American drug market have contributed to alarming rates of fentanyl-related overdose deaths (2).

The use of non-medical fentanyl and its analogues has changed drastically over the last ten years. (3) In the 2000s and the early years of non-medical fentanyl use, fentanyl was diverted from clinical settings, mostly in the form of transdermal patches. Fentanyl patches were “cooked into fentanyl tea” and the fluid was injected intravenously along with extracted fentanyl. (4) This was a rare occurrence, and the practice was only found in parts of Europe (e.g., in Germany). A decade later, fentanyl made its way into the street drug market. Due to its synthetic quality, high availability, and lower cost, fentanyl was commonly mixed into other desired substances to offset the cost for producers and sellers. (3) Originally a contaminant, fentanyl has now become increasingly present in street opioids, stimulants and hallucinogens. (5) Over time, people who use drugs (PWUD) have also become increasingly accustomed to fentanyl added to other illicit substances. Based on legally available precursors, fentanyl has also increasingly been produced in local private laboratories. (3) Combined with a lower cost of production, increased availability in the illicit drug market, and the rapid, intense onset of effect, these attributes have altered fentanyl’s identity from an unwanted contaminant to a desirable drug of choice. (2) However, the demographic characteristics and comorbidities common to individuals who prefer fentanyl to other substances and use it intentionally as their drug of choice are unknown in the current literature. Intentional fentanyl use has been defined in this review as seeking fentanyl in the illicit drug market or using substances that are known to contain fentanyl; in other words, having fentanyl as one’s drug of choice, seeking out fentanyl, and not using fentanyl by accident. The details around patterns of use and motivation to use are also currently unknown.

This systematic review aimed to evaluate the evidence on intentional fentanyl use among PWUD by summarizing demographic variance, reasons for use, and resulting patterns of use to inform the development of effective interventional approaches and settings and identify critical research questions.

## 2 Methods

### 2.1 Review protocol

The Preferred Reporting Items for Systematic Review and Meta-Analysis (PRISMA) guidelines were used to ensure the details in the methodology is comprehensive (6). A protocol for this review has been registered with PROSPERO (registration number: CRD42021272111) (7). Research ethics board review was not required as this study relies exclusively on publicly available information that is legally accessible to the public.

### 2.2 Search strategy

The search strategy in this study was developed with a combination of free text keywords and MeSH and non-MeSH keywords. Search items were adapted with database-specific filters. Four different databases: Ovid MEDLINE (1860-May 2021); Embase (1952-May 2021); Web of Science (1900-May 2021); and PsychINFO (1900-May 2021). The search strategies for Ovid MEDLINE, Embase, Web of Science, and PsychINFO are provided in the supplemental material (**Supplementary Tables S1A-D**). References of all included papers were hand-mined, and any additional documents were added from gray literature such as from thesis dissertations and Google Scholar. The last search was completed on May 29, 2021.

### 2.3 Study inclusion and exclusion criteria

Studies were included if they reported data on the intentional use of non-medical fentanyl or any fentanyl analogues in PWUD older than 13. The term PWUD refers to those who use illicit drugs or use prescription drugs non-medically. Papers from all regions of the world were considered as long as they were written or were available in the English language. Only peer-reviewed original articles were included, including case reports/series. Letters, reviews, meta-analyses, toxicology or coroner’s reports, commentaries, and editorials were excluded. Studies that included non-human participants, did not mention explicit intentional fentanyl use, or only discussed medical indications for fentanyl were excluded. Control or other comparison groups were not relevant to this study and the outcome of interest was intentional fentanyl use.

### 2.4 Screening and data extraction

**Figure 1:**
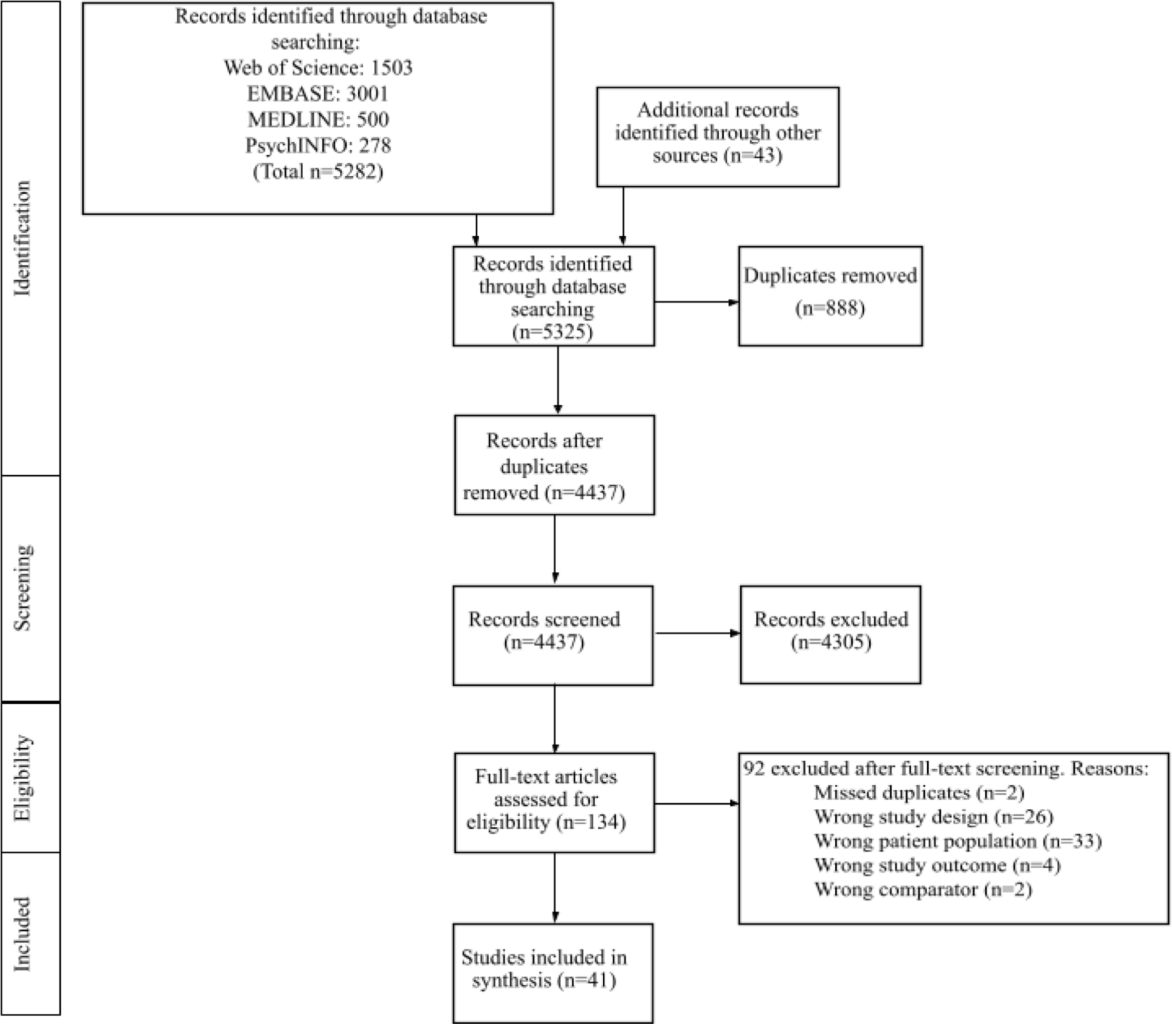
Flow of literature search. (MEDLINE, Medical Literature Analysis and Retrieval System Online; EMBASE, Excerpta Medica database) Legend. Wrong comparator: original study had not included a group or population with intentional fentanyl use for comparison or analysis within the study text.

The PRISMA flow diagram was used to review selected articles in sequential fashion (**Figure 1**). Titles and abstracts of studies retrieved using the search strategy were screened by at least two of five reviewers (VWLT, JSHW, JNW, HF, NR). Any inconsistencies were reviewed by a third reviewer (VWLT or JSHW). The inclusion and exclusion criteria were strictly adhered and all articles were independently screened to minimize bias. Full text documents were independently assessed by at least two of five reviewers (VWLT, JSHW, JNW, HF, NR) for inclusion and any disagreements were resolved by consensus. A standardized table with predetermined categories was used for independent data extraction by at least two reviewers. Data on patient demographics, study setting, study methods, motivations for drug use, patterns of use, and associated attributes or behaviours of participants were collected.

### 2.5 Study quality assessment

Quality synthesis and evaluation of bias for article inclusion was completed in alignment with

the Newcastle-Ottawa Quality Assessment scale for cohort and case-controlled studies (8). Cross-sectional studies were evaluated with the modified Newcastle-Ottawa Quality Assessment scale for comparable results (9). Qualitative studies were appraised with the Critical Appraisal Skills Program (CASP) checklist (10). For case studies and series, we noted the following criteria for assessing methodological quality: timeline of recruitment, prospective or retrospective recruitment. Meta-analyses and reviews were not included in this study and there were no randomized controlled trials found.

### 2.6 Analysis

Any inconsistencies were brought up to VWLT for review and final decision. The approach for analysis was conducted by separation of studies into three categories: studies discussing intentional fentanyl use with other substance use but where data were not distinguishable, studies with intentional fentanyl use only, and studies comparing intentional fentanyl using cohorts with non-intentional fentanyl using cohorts. This was conducted by three reviewers (NR, JSHW, JNW) with a second reviewer for each grouped analysis. Details of interest captured for each category of studies include demographic descriptors such as mean age, gender distribution, racial background, and socioeconomic status. Other relevant details captured include years of substance use, substance use patterns, overdose history, motivation for substance use, and usage patterns. In order to calculate pooled means for age, we estimated means from studies which only reported medians by using Luo et al., 2018’s model (11, 12).

## 3 Results

The search resulted in 4437 studies after de-duplication, 132 were selected for full-text review, and 41 were included (**Figure 1**). Of the 41 studies included, 23 were in the United States (13–35); seven in Canada; (36–42) two in Sweden; (43, 44) one each in Australia, (45) Germany, (46) Denmark, (47) France, (48) Estonia, (49) the UK, (50) and Turkey. (51) Two studies were done online with no note of specific country involvement (**Table 1**) (52, 53). A mean of 62·63% of participants were male and 64·05% were White. The mean of average age was 41·36 (SD=10·86).

**Table 1:** Study details for all included articles (N=41).

	Sample size (N)	How did study determine intentional fentanyl use	Separation possible?	Recruitment method	City, country	Timelines	Methodology of study	Method of data analysis
Comprehensive Description of Studies Involving Intentional Fentanyl Use That is Undistinguishable from Other Substance Use (N=19)								
Amlani et al. (36)	242	Self-reported intentional use	No	HR services across BC	British Columbia, Canada	Feb - Mar 2015	Cross-sectional linking demographics & substance usage patterns with urine tests	Descriptive statistics
Bach et al. (15)	165 (survey), 129 (urine samples)	Survey	No	Triage screening of patients for substance use	Baltimore, Maryland, USA	May - July 2018	Cross-sectional study, urine analysis & survey	Urine analysis
Balsamo et al. (52)	86,445	Information provided on Reddit comments	No	Publicly available Reddit data set	Online - Reddit	2014- 2018	Semi Automatic information retrieval algorithm Reddit	Statistical modeling
Bardwell et al. (37)	21	Interviews	No	Recruited from two cohort studies	Vancouver, Canada	Dec 2019- Mar 2020	Qualitative semi-structured interviews	Thematic coding
Buresh et al. (16)	994	Self-reported intentional use	Yes	SSP & HIV treatment services, community outreach	Baltimore, Maryland	Nov 2017- June 2018	Cross-sectional study quantitative survey	Standard descriptive statistic
Ciccarone et al. (13)	38	Qualitative interview	No	Recruited during daily activities	Massachusetts & Hampshire	June 2016	Qualitative rapid study & semi-structured interview	Analytic memos, inductive analysis
Daniulaityte et al. (18)	60	Self-reported heroin/NPF use	No	Online & community outreach	Dayton, Ohio, US	May 2017-Jan 2018	Semi structured interviews & urine drug screen	Descriptive statistics
Gryczynski, et al. (19)	1,174 (review records), 114 (anonymous surveys)	Survey	No	Convenience sampling of outpatients	Baltimore, Maryland, US	2018	Review of treatment records & anonymous survey	Descriptive statistics & logistic regression model
Kenney et al. (21)	231	Interview	No	Patients seeking opioid withdrawal management	Fall River, Massachusetts	Apr - Sept 2017	Survey	Quantitative, descriptive, inferential statistics
Krause et al. (46)	960 (UDS), 401 (questionnaire)	Questionnaire	No	Recruited from outpatient clinics	Munich, Germany	2008 - 2012	Cross sectional- Urine analysis & questionnaire	Quantitative
McLean et al. (27)	125 (surveys), 30 (interviews)	Interviews	No	Targeted sampling, advertised in local drug treatment clinics & community	Allegheny, Fayette, Greene, Washington	July 2017 - July 2018	Qualitative interview & surveys	NVivo, descriptive statistics
Moeller et al. (44)	24 threads with 8761 posts	Some of the users purchased fentanyl analogs wittingly	N/A	Online through flashback.org	Sweden	Sept 2012-July 2019	Thematic analysis of a public internet forum Flashback	Thematic analysis
Nolte et al. (30)	589 (survey), 22 (in-depth interview)	Interviews	No	Street outreach, HR agencies & referrals	Rural Northern New England, Northeastern United States,	May 2018 – Oct 2019	Interview & survey	Descriptive statistics & thematic analysis
O'Rourke et al. (31)	373	Cross-sectional survey	No	Recruited PWID from a SSP & in community locations	Cabell County, West Virginia USA	June - July 2018	Cross sectional survey with statistical analysis	Descriptive statistics
Park et al. (32)	326	Survey	No	Targeted sampling at SSP & HR services.	Baltimore, Maryland; Boston, Massachusetts; & Providence, Rhode Island	June – Oct 2017	Survey, interview	Descriptive statistics & logistic regression analysis
Silverstein et al. (33)	63	N/A	No	Community outreach	Dayton, US	May 2017- June 2019	Qualitative interviews	NVivo, thematic coding
Uuskula et al. (49)	110	Interviewer-administered questionnaire	No	Respondent-driven sampling	Tallinn, Estonia	2009 to 2013	Cross-sectional & observational	Quantitative analysis
Wallace et al. (41)	187	Self-reported intentional use	No	Convenience sampling from sites distributing clean injecting supplies	Victoria, Canada	June - Sept 2016	Cross-sectional survey	Logistic regression analysis
Weicker et al. (35)	20	Interviews	No	Street outreach in targeted locations	Baltimore US	Oct 2018 - Dec 2019	Qualitative interviews	MAXDQA via priori & inductive codes
Comprehensive Description of Studies Involving Intentional Fentanyl Use Only (N=13)								
Eiden et al. (48)	1	Self-reported intentional use	N/A	Patient admitted to emergency department	France	May 2016	Case report	Descriptive statistics
Firestone et al. (38)	25	Qualitative interviews	No	With the help of community service provider & peer contacts	Toronto, Ontario, Canada	Mar- June 2007	Exploratory (interview-based) qualitative study	N/A
Gecici et al. (51)	1	Self-reported intentional use	Yes	Inpatient hospital admission	Turkey	2010	Case report	Case report
Guerrieri et al. (43)	40	Witnesses, police findings	No	Report of a series of forty fatal intoxications	Sweden	Apr-Oct 2016	Femoral blood analysis & case studies	Analyst1 1.6.2 software
Gunn et al. (20)	21	Interviews	N/A	Flyers & referrals at local SSP, community outreach services & primary care practices	Boston, MA, United States.	May-Nov 2018	Qualitative interviews	NVivo, deductive & inductive thematic analysis
Kilwein et al. (22)	122	Questionnaire (descriptive survey)	No	Online postings	34 US states	Feb 2016 - Apr 2017	Descriptive study, fentanyl questionnaire	N/A
Kimergard et al. (47)	14	Medical history during intake as a patient	N/A	Outpatients seeking treatment for fentanyl smoking from an addiction service	Southern Denmark	Aug- Dec 2015	Retrospective case review	N/A
Lyttle et al. (50)	N= 1	Applied 5 patches to end her life	Yes	N/A	Bristol, UK	N/A	Case report	Case report
Marquardt et al. (25)	1	Observed by paramedics	No	Man seen by paramedics	Sacramento, California, United States	1994	Case report	Descriptive statistics
Mrvos et al. (29)	76	Ingested intact fentanyl patches	Yes	Three RPIC medical record databases	Pittsburgh, USA	2000 & 2008	Retrospective case review	Descriptive statistics
Reeves et al. (53)	1	Injected content of transdermal patch	No	Inpatient hospital admission	N/A	2002	Case report	N/A
Tharp et al. (34)	4	Transdermal patch use & injected	No	Post-mortem analysis	North Carolina, USA	Jan 1997 - July 2001	Post-mortem analysis	N/A
Woodall et al. (42)	7	Witness reports & autopsy	No	Identified via a retrospective analysis of fentanyl-related deaths	Ontario, Canada	Jan 2002 - Dec 2004	Post-mortem autopsy, blood analysis & toxicological findings	Toxicological analysis
Comprehensive Description of the Intentional Fentanyl Using Subpopulations Among Studies Comparing Intentional Fentanyl with Non-intentional Fentanyl (N=9)								
Antoine et al. (14)	4	Self-reported intentional use	Yes	Participants were part of RCT of a sleep medication during opioid tapering	Not clear what city, but all authors from Maryland; United States	2021	Case series	Descriptive statistics
Chandra et al. (17)	104	Self-reported intentional use	Yes	Recruited from an addiction treatment setting using clinic-based advertisements & community	New Haven, Connecticut, US	July 2018 - Oct 2019	Cross-sectional- survey using an audio computer-assisted self-interview	Multivariable logistic regressions, descriptive statistics
Geddes et al. (45)	2378	Survey	N/A	The annual Australian NSP survey	Australia	2014	Cross sectional - self-administered questionnaire & antibody testing.	Descriptive statistics & logistic regression models
Karamouzian et al. (39)	303	Self-reported intentional use	Yes	Recruited from HR sites	British Columbia, Canada	May - Aug 2018	Cross-sectional study, & urine sample & survey	Multinomial logistic regression models
Kline et al. (23)	432	Survey	No	Methadone maintenance, acute residential detoxification programs	New Jersey, USA	Oct 2018 - Mar 2019	cross-sectional quantitative study	Descriptive statistics
Macmadu et al. (24)	199	Self-reported intentional use	Yes	Targeted canvassing, snowball sampling & online	Rhode Island, United States	Jan 2015 - Feb 2016	Cross-sectional study with interviews & surveys.	Descriptive statistics & logistic regression models
Mazhnaya et al. (26)	311	Survey	Yes	Purposive sampling at the HR program & community	Cabell County, WV	June- July 2018.	Audio computer-assisted self-interview (ACASI)	Descriptive statistics, Kolmogorov- Smirnov, regression
Mitra et al. (40)	578	Self-reported intentional use	Yes	Self-referral & community outreach	Vancouver, Canada	Dec 2016 Nov 2017	Questionnaire	Descriptive statistics, logistic regression model
Morales et al. (28)	308	Self-report via survey	Yes	Convenience sampling at HR organizations	Baltimore, Boston, Massachusetts, & Rhode Island	June – Oct 2017	Cross sectional surveys	Descriptive statistics

NPF, non-pharmaceutical fentanyl; PWU, people who use; SSP, syringe service programs; HR, harm reduction; UDS, urine drug screening; HIV, human immunodeficiency virus; N/A, not applicable.

Outcomes were analyzed in three groups. Nineteen studies discussed intentional fentanyl use with other substance use where data were not distinguishable (**Table 2**), 13 studies discussed intentional fentanyl use only (**Table 3**), and 9 studies compared intentional fentanyl using cohorts with non-intentional fentanyl using cohorts (**Table 4**). It should be noted that there was little data to extract from studies examining intentional fentanyl use only, as 11 of the 13 studies were case reports/series. Moreover, of the 11 case reports/series, 3 were post-mortem analyses, all of which reported illicit intentional use of fentanyl. (34, 42, 43) More comprehensive details are available as online supplements (**Supplementary Tables S2**–**S4**).

**Table 2:** Comprehensive description of studies involving intentional fentanyl use that is undistinguishable from other substance use (N=19).

	Sample Size	Age (Years)	Gender (% Male)	Overdose history	Race/Ethnicity	Socioeconomic factors
Amlani et al. (36)	242	Range 19 – 29: 19% 30 – 39: 28% 40 – 49: 34% 50+: 19%	58%	Overdose within last month: 10% Overdose within last week: 2%	N/R	N/R
Bach et al. (15)	165	Estimated Mean (SD): 47.95 (14.13) Median (IQR): 49 (38 – 57)	77.00%	Opioid overdose: 42 (25.5%)	N/R	N/R
Balsamo et al. (15)	86,445	N/R	N/A	N/A	N/A	N/A
Bardwell et al. (37)	21	Estimated Mean (SD): 48.3 (7.4) Range: 35 – 63 Median: 48	57.14%	N/R	White: 12 Indigenous: 9	Employment - Income generation (last 30 days) Social assistance: 21 Drug selling: 16 Recycling/vending: 13 Part-time employment: 10 Theft: 8 Panhandling/busking: 5 Sex work: 1
Buresh et al. (16)	994	Mean (SD): 55 (9.1)	65%	Reported a recent non-fatal overdose (any drug): 35 (4%)	African American: 84%	Education - 46% completed high school Employment – employed: 14% Employment – income <$5K: 70% Medical history - 31% HIV positive Housing - 10% reported homelessness in the prior 6 months Relationship - 48% ever married
Ciccarone et al. (13)	38	Range: 19 - 52	60.52%	N/R	Of those stating their ethnicity: White: 16 African American: 3 Hispanic: 10 Mixed ethnicity: 7	N/R
Daniulaityte et al. (18)	60	Mean (SD): 39 (9.5)	48.3%	Mean unintentional drug-related overdoses in their lifetime: 2.8 Self-perceived risk of overdose as high: 11.7% Self-perceived risk of overdose as moderate: 33%	White: 91.7% African American: 6.7% Other: 1.7%	Education - High school education or less: 70% Education - High school or GED: 36.7% Education - some college or more: 31.7% Employment - Unemployed: 75%
Gryczynski et al. (19)	1,174	Mean (SD): 40.7 (11.4)	65.50%	N/R	Black/African-American: 59.6% White: 40.4%	Relationship - 5.5% married Relationship - 94.5% not married Medical history - 58.7% current mental health diagnosis
Kenney et al. (21)	231	Mean (SD): 34.0 (9.2)	73.20%	Ever overdosed: 127 (55.0%)	White: 193 (83.6%) Black: 6 (2.6%) Other: 32 (13.0%) Latino: 27 (11.7%)	Education - mean years education: 12.0 (±1.7)
Krause et al. (46)	960 (UDS) 401 (questionnaire)	Range: 18 - 30: 19.4% 30 - 40: 41.1% >40: 39.5%	64.60%	N/R	N/R	N/R
McLean et al. (27)	125	Mean (SD): 34.5 (8.7) Range: 20 – 62	66.4%	Overdosed and needed medical intervention to be revived: Yes, once: 13 Yes, more than once: 19	Non-Hispanic White: 80% Non-Hispanic Black: 6.7%	Education - 10% didn’t complete high school Education - 43.3% completed high school Employment - 56.7% unemployed
Moeller & Svensson (44)	24 threads with 8761 posts on Flashback.org	N/A	N/R	N/R	N/R	N/R
Nolte et al. (30)	589	N/R	58.7%	N/R	White: 90.3% Non-Hispanic: 95.2%	N/R
O'Rourke et al. (31)	373	Mean (SD): 35.8 (8.6)	59.50%	In the past 6 months: Experienced a drug overdose 43.7%	White, non-Hispanic: 83.4%	Education - had at least a high school education: 71.7% Relationship - either married or in a relationship: 47.3% Housing - consider themselves homeless: 57.1% Arrest - reported been recently arrested: 30.6%
Park et al. (32)	326	Range: < 35: 23.9% ≥ 35: 76.1%	59.1%	Had a history of overdose: 64% Overdosed more than once: 34.7%	Non-White: 64%	Education - 39% < high school Housing - 68.7% currently homeless Employment - 86.8% unemployed Employment - 57% had sold drugs in the past 3 months Incarceration - history of arrest 47% Arrest - 46.5%
Silverstein et al. (33)	63	Mean (SD): 38.9 (10.6) Range: 19 - 70	54%	N/R	Non-Hispanic White: 85.7% African American: 12.7% Hispanic: 1.6%	Education - Less than secondary: 22.2% Education - Secondary school degree: 27% Education - Some college or tech school: 38.1% Education - Post secondary: 12.7% Housing - Shelter: 4.8% Housing – Streets: 1.6%, Employment - Employed full time: 12.7% Employment - Part time: 20.6% Employment - Unemployed: 47.6% Employment - Unemployed due to disability: 15.9%
Uuskula et al. (49)	110	Mean (SD): 24.5 (7.5)	69.10%	N/R	N/R	Education - 52% had education (10+ years) Employment - 37% were employed Incarceration - 25% have been in prison Medical history - 19% HIV
Wallace et al. (41)	187	Estimated Mean (SD): 40.4 (12.7) Median (IQR): 40 (32 – 49)	64.7%	Overdosed at least once in the previous 6 months: 56 (29.9%)	White: 62%	N/A
Weicker et al. (35)	20	Estimated Mean (SD): 37.4 (9.9) Range: 20-57 Median: 37	45%	N/R	Black:10 White: 9 Multiracial: 1	Housing – homeless: 75%

UDS, urine drug screening; HIV, human immunodeficiency virus; SD, standard deviation; N/R, not reported; N/A, not applicable.

**Table 3:** Comprehensive description of studies involving intentional fentanyl use only (N=13).

	**Age (years)**	**Gender (% male)**	**Race/Ethnicity (% White)**	**Substance use patterns**	**Overdose history**	**Socioeconomic factors**
Eiden et al. (48)	59	100%	N/R	Transmucosal fentanyl use, 5 to 15 cigarettes/day: 100%	N/R	N/R
Firestone et al. (38)	Range: 18 - 50	60%	N/R	Participants used a “variety of other drugs” but they were not specified.	N/R	N/R
Gecici et al. (51)	59	100%	100%	History of cannabis abuse for 20 years, but stopped 10 years ago: 100% Use of transdermal fentanyl patches 3-4 times per day: 100%	N/R	Had 3 children. Was a driver but has not worked in the last 1.5 years.
Guerrieri et al. (43)	Mean (SD): 32.05 (9.49) Range: 18 - 53	85%	N/R	Acrylfentanyl was identified along with other drugs: 97.5% No other drugs but acrylfentanyl were found: 2.5% 5 cases were discussed more extensively: fentanyl nasal spray (3/5), fentanyl tablets (2/5)	Fatal accidental OD: 85% Possibly suicide: 15%	N/R
Gunn et al. (20)	18 – 25 (n = 10) 35+ (n = 11)	52%	100% English Speaking	9.5%: actively seeking fentanyl 42.9%: passive use of fentanyl (doesn't seek it) 47.6%: does not want to use fentanyl	History of at least 1 OD: 95.2%	N/R
Kilwein et al. (22)	Mean (SD): 32.32 (10.28) Range: 18 - 67	46%	71.3%	Lifetime history of other illicit drug use: 94% Lifetime history of nonmedical use of another opioid: 73.8%	N/R	Some high school education: 4.9% A high school diploma/General Education Diploma (GED): 35.2% A trade/technical degree: 12.3% Some college education: 28.7% A bachelor’s degree: 14.8% A graduate/professional degree: 4.1% Enrolled in college: 18.9%
Kimergard et al. (47)	Mean (SD): 27.9 (4.7) Range: 23 - 37	93%	N/R	Cannabis: 88.9% Other opioids/metabolites, including codeine, morphine, oxycodone & oxymorphone: 66.7% Cocaine: 44.4% Amphetamine: 33.3%	N/R	N/R
Lyttle et al. (50)	15	0%	N/R	N/R	OD & attempted suicide by use of fentanyl patches: 100%	N/R
Marquardt et al. (25)	34	100%	N/R	Inhalation of fentanyl patch: 100%	N/R	N/R
Mrvos et al. (29)	Mean: 32.6 Range: 15 - 56	59.20%	N/R	Ingestion of whole fentanyl patches: 100%	N/R	N/R
Reeves et al. (53)	35	0%	N/R	History of IV drug use: 100%	Death by OD: 100%	N/R
Tharp et al. (34)	Mean (SD): 38.5 (2.89) Range: 35 - 42	100%	100%	History of drug use: 75% No known history of drug use: 25%	Fatal fentanyl OD, suicide: 25% Fatal fentanyl poisoning, accidental: 50% Fatal fentanyl toxicity, accidental: 25%	N/R
Woodall et al. (42)	Mean (SD): 39.14 (10.21) Range: 20 - 51	57%	N/R	Most had a history of drug abuse	Fatal fentanyl OD: 28.6% Fatal fentanyl & ethanol OD: 42.9% Mixed drug intoxication death: 14.3% Death due to fentanyl & medical causes: 14.3%	N/R

IV, intravenous; N/A, not applicable; N/R, not reported; OD, overdose; SD, standard deviation.

**Table 4:** Comprehensive description of the intentional fentanyl using subpopulations among studies comparing intentional fentanyl and non-intentional fentanyl use (N=9).

	Intentional fentanyl use group						Non-intentional fentanyl use group					
	Group	Age (years)	Gender (% male); Ethnicity (% Caucasian)	Substance use patterns	Overdose	Socioeconomic	Comparison group	Age (years)	Gender (% male); Ethnicity (% Caucasian)	Substance use patterns	Overdose	Socioeconomic
Antoine et al. (14)	Intentional fentanyl use (n=3)	18-25: 33% (1/3) 26-40: 33% (1/3)41-55: 33% (1/3)	100%; 100%	Heroin/fentanyl use intranasal: 100% (3/3) Prescription opioid misuse: 33% (1/3) Cocaine use: 66% (2/3)	N/R	N/R	Non-intentional fentanyl use** (n=1)	26‐40: 100% (1/1)	0%; 0%	Heroin/fentanyl use intravenous/intranasal: 100% (1/1)	N/R	N/R
Chandra et al. (17)	Purposeful fentanyl use (n=45)	Mean (SD): 37.5 (8.8)	64.4%; 82.2%	Heroin: 86.7% Cocaine: 88.9% Poly drug: 91.1% Inject daily: 28.9%	Non-fatal OD in past year: 28.9%	High school graduate: 75.6% Income level < $10,000: 68.9% Currently married/living with partner: 28.9% Homeless in past year: 60.0%	No purposeful fentanyl use (n=59)	Mean (SD): 43.1 (9.3)	49.2%; 74.6%	Heroin: 15.3% Cocaine: 78.9% Poly drug 85.6% Inject daily: 18.3%	Non-fatal OD in past year: 11.9%	High school graduate: 67.8% Income level <$10,000: 78.0% Currently married/living with partner: 23.7% Homeless in past year: 47.5%
Geddes et al. (45)	Recent fentanyl injection (n=193)	<30 years: 12% 30–39 years: 43% 40–49 years: 33% >49 years: 12%	75%; 77%	Fentanyl as the main opioid in last 6 months: 78% Heroin as the drug last injected: 32% Currently in OST: 37% Daily injection: 78%	Overdose in last 12 months: 37%	HCV negative: 38% HCV positive: 62%	No recent fentanyl injection (n=655)	<30 years: 12% 30–39 years: 37% 40–49 years: 32% >49 years: 19%	69%; 84%	Currently in OST: 34% Not currently in OST: 62% Daily injection: 61%	Overdose in last 12 months: 21%	HCV negative: 38% HCV positive: 62%
Karamouzian et al. (39)	Known use (n=117)	≥50: 22.2%40–49: 32.1%30–39: 52.2% 19–29: 48.33%	38.7%; N/R	Cannabis: 39.2% Methadone: 64.1% Heroin/morphine: 61.8% Oxycodone: 85.7% Crystal meth: 55.3% Cocaine: 57.6% Crack: 52.3% Benzodiazepine: 51.4% Polydrug: 66.4% Preferred ROA injection: 55.5%	Experienced non-fatal overdose in the last 6 months: 51.8%	Unstable housing (current): 52.3% Paid employment: 27.2% Medium/large urban cities: 48.1% Small urban/rural communities: 22.3%	No fentanyl use (n=120)	≥50: 66.6%40–49: 33.3% 30–39: 26.1% 19–29: 36.6%	42.3%; N/R	Cannabis: 47.7% Methadone: 20.5% Heroin/morphine: 9.0% Oxycodone use: 14.2% Crystal meth: 24.4% Cocaine: 28.8% Crack: 35.3% Benzodiazepine: 34.2% Polydrug: 13.1% Preferred ROA injection: 16.8%	Experienced non-fatal overdose in the last 6 months: 22.2%	Unstable housing (current): 31.4% Paid employment: 56.3% Medium/large urban cities: 25.6% Small urban/rural communities: 63.39%
Kline et al. (23)	Persistent overdose subgroup* (n=40)	Mean (SD): 38.03 (11.49)	72.5%; 69.2%	Often/always mixes opioids with one or more other drugs: 70% Heavy alcohol use: 40% IV Injection: 72.5%	Mean number of overdoses: 8.03	Homeless: 15%	No lifetime overdoses (n=238)	Mean (SD): 41.02 (11.72)	48.3%; 46.4%	Often/always mixes opioids with one or more other drugs: 51.3% Heavy alcohol use: 17.2% IV Injection: 28.2%	Mean number of lifetime overdoses: N/A	Homelessness: 10.1%
Macmadu et al. (24)	Self-report intentional use of FCH in prior 6 months (n=22)	Median (IQR): 27 (25–28)	72.7%; 95.5%	Heroin: 81.8% NMPO: 63.6% Cocaine: 36.4% Non-medical benzodiazepine: 59.1% Diverted pharmaceutical fentanyl: 72.7% Injection drug use: 40.9%	Ever experienced a non-fatal overdose: 63.6%	Education beyond high school: 45.5% Ever detained in jail: 59.1% Ever homeless: 77.3% Mental health diagnosis: 86.4% Ever HCV positive: 33.3%	No FCH use in prior 6 months (n=177)	Mean (IQR): 24 (22–27)	64.4%; 57.1%	Heroin: 18.6% NMPO: 45.2% Cocaine: 7.9% Non-medical benzodiazepine: 26.6% Diverted pharmaceutical fentanyl: 6.8% Injection drug use: 9.0%	Ever experienced a non-fatal overdose: 22.0%	Education beyond high school: 50.9% Ever detained in jail: 45.8% Ever homeless: 51.4% Mental health diagnosis: 71.2% Ever HCV positive: 8.3%
Mazhnaya et al. (26)	Prefer drugs containing fentanyl (n=135)	Median (IQR): 35 (28–40)	48.9%; 84.4%	Injection drug use past 6 months Fentanyl: 83.7% Heroin: 97.8% Buprenorphine or Buprenorphine/Naloxone: 25.2% Painkillers: 25.2% Crystal meth: 76.3% Speedball: 55.6% Cocaine: 49.6% Other drugs use past 6 months: Smoked heroin: 23.7% Swallowed fentanyl: 17.8% Swallowed painkillers: 34.8% Swallowed Buprenorphine or Buprenorphine/Naloxone: 30.4%	Number of overdoses experienced in past 6 months 0 :41.5% 1-2: 29.6% 3-5: 15.6% 5+: 13.3%	High school graduate: 71.6% Single: 55.2% Sexual Minority: 17.2% Homeless: 60% Unemployed: 71.9% Food insecurity: 68.9% Transactional sex work in past 6 months: 68.9% Arrested in past 6 months: 37.8%	Do not prefer drugs containing fentanyl (n=176)	Median (IQR): 37 (31–42)	67.1%; 93.8%	Injection drug use, past 6 months: Fentanyl: 57.4% Heroin: 86.9% Buprenorphine or Buprenorphine/Naloxone: 29.6% Painkillers: 22.2% Crystal meth: 74.3% Speedball: 35.2% Cocaine: 30.7% Other drug use past 6 months: Smoked heroin: 13.1% Swallowed fentanyl: 5.7% Swallowed painkillers: 28.4% Swallowed Buprenorphine or Buprenorphine/Naloxone: 31.3%	Number of overdoses experienced in past 6 months 0: 53.4% 1-2: 25.6% 3-5: 12.5% 5+: 8.5%	High school graduate: 72.2% Single: 49.4% Sexual Minority: 13.1% Self-homeless: 56.3% Unemployed: 62.5% Food insecurity: 65.3% Transactional sex work in past 6 months: 11.4% Arrested in past 6 months: 35.8%
Mitra et al. (40)	Self-reported intentional fentanyl users (n=386)	Median (IQR): 39 (28.2-50.4)	66.1%; 52.5%	Heroin: 60.9% Prescription opioid: 3.6% Stimulant: 43.5% OAT: 58.8% Injection drug use: 87%	High/moderate perceived risk of fentanyl overdose (men; women): 50.4%; 40.5%	DTES residency: 63.0% Incarceration: 9.9% Exchanged money for sex: 11.4% Experienced violence: 13.6%	Self-reported unintentional fentanyl exposure (n=192)	Median (IQR): 44.7 (34.6-53.4)	54.2%; 50.0%	Heroin: 43.2% Prescription opioid: 5.7% Stimulant use: 41.7% OAT: 55.7% Injection drug use: 80.7%	High/moderate perceived risk of fentanyl overdose (men; women): 52.0%; 62.5%	DTES residency: 73.0% Incarceration: 8.3% Exchanged money for sex: 12% Experienced violence: 14.6%
Morales et al. (28)	Preference for fentanyl (n=83)	Median (IQR): 38 (32–46)	62.7%; 59%	Prescribed opioid: 53% Medication-assisted treatment: 75% Daily drug use: 91.6% Heroin injection: 78.3% Heroin, smoked/snorted: 32.5% Crack cocaine use: 73.5% Snorted cocaine: 19.3% Cocaine injection: 44.6% Speedball injection: 53% Injection drug use: 83.1%	Never: 25.3% More than a year ago: 31.3% Within the last year: 43.4% Suspected due to fentanyl: 86.1%	Currently homeless: 69.9% Main sources of income last 3 months illegal work: 63.9% Arrested / incarcerated, last year: 59.0%	Does not prefer fentanyl (n=225)	Median (IQR): 45 (37–52)	59.1%; 29.3%	Prescribed opioid use: 53.3% Medication-assisted treatment: 70% Daily drug use: 75.6% Heroin injection: 64% Heroin, smoked/snorted: 51.1% Crack cocaine use: 69.3% Snorted cocaine: 28% Cocaine injection: 35.1% Speedball injection: 42% Injection drug use: 68.4%	Never: 37.3% More than a year ago: 17.3% Within the last year: 45.3% Suspected due to fentanyl: 91 (89.2%)	Currently homeless: 68.0% Main sources of income, last 3 months illegal work: 41.3% Arrested / incarcerated, last year: 42.2%

*Significant finding that this subgroup would more likely take fentanyl intentionally.

**Self-reported heroin use; UDS came back positive for fentanyl.

N/R, not reported, N/A, not applicable; FCH, fentanyl-contaminated heroin; DTES, downtown east side; OAT, opioid agonist treatment.

### 3.1 Demographics

Regarding age among studies comparing intentional fentanyl using cohorts with non-intentional fentanyl using cohorts, participants were a pooled mean of 37·65 years (SD=13·77) in the intentional fentanyl-using cohort compared to 38·89 years (SD=10·53) in the non-intentional fentanyl-using cohort. This compared with 32·32 years (SD=9·73) in studies examining intentional fentanyl use only, and 43·34 years (SD=10·34) in studies examining intentional fentanyl use with other substance use.

Regarding gender distribution, studies comparing the two groups reported a mean of 62·52% male participants in the intentional fentanyl-using cohort and 60·54% in the non-intentional fentanyl-using cohort. This compares with 58·93% in studies on intentional fentanyl use only and 63·64% in studies on fentanyl with other substance use. Only one study comparing the two groups directly reported non-binary or gender non-conforming prevalence, which was 37·5% in both the fentanyl using cohort and non-intentional fentanyl using cohort (39). There were no mentions of non-binary or gender nonconformity in the fentanyl use only studies, and one study in the studies on fentanyl with other substance use (31).

Among studies comparing intentional fentanyl using cohorts with non-intentional fentanyl substance using cohorts, individuals who intentionally use fentanyl were more likely to be male and young (17, 23, 28, 40). In Krause et al., 2017, it was reported that a significant difference was found between younger age and fentanyl consumption (p=0·003) (46). In contrast, self-reported unintentional exposure to fentanyl was positively associated with women and older age (40, 49).

Elaborating on racial differences, studies comparing the two cohorts reported a pooled mean of 66·47% participants who were White in the intentional fentanyl cohort and 65·74% participants who were White in the non-intentional fentanyl cohort. This compared with a pooled percentage of 72·43% in studies with intentional fentanyl use only and 62·12% in studies on fentanyl with other substance use. In a study from Baltimore, Boston, and Providence, fentanyl preference was associated with non-Hispanic white race among PWUD (N=308) (32). Similarly, from a case-series that describes buprenorphine/naloxone inductions of four individuals who tested positive for fentanyl, three intentional-using individuals were male and White, while the unintentionally-using individual was female and non-White (14). One study further showed that African American respondents were less likely to report having ever used fentanyl (16). Only one study reported the opposite - that participants preferring drugs containing fentanyl were less likely to be White and non-Hispanic (26).

### 3.2 Socioeconomic considerations

Among the three groups of studies, each group reported at least one study with either unemployment or educational attainment as a socioeconomic factor. Unemployment was considered as a socioeconomic factor in seven out of 19 studies where intentional fentanyl use and other substance use was not distinguishable (**Table 2**) (16, 18, 27, 32, 33, 37, 49). The only studies of the 13 that reported on intentional fentanyl use only was a case report that included unemployment as a socioeconomic factor (**Table 3**) (51). Three of nine studies comparing intentional fentanyl and non-intentional fentanyl using cohorts reported unemployment or illegal work as main source of income as being more common with the intentional fentanyl using group (**Table 4**) (26, 28, 39).

Educational attainment was reported in eight of 19 studies which did not distinguish fentanyl use and other substance use (**Table 2**) (16, 18, 21, 27, 31–33, 49). Three studies in the group comparing intentional fentanyl using cohorts with non-intentional fentanyl using cohorts (**Table 4**) and one study in the intentional fentanyl use only group (**Table 3**) reported educational attainment as a socioeconomic characteristic (17, 22, 24, 26). In Macmadu et al., 2017, the group of individuals with intentional fentanyl-contaminated heroin use also had a lower proportion who had attained education beyond high school (24).

Among six studies in the group comparing intentional fentanyl using cohorts with non-intentional fentanyl using cohorts, it was reported that individuals who intentionally used fentanyl were more commonly homeless and experiencing unstable housing (**Table 4**) (17, 23, 24, 28, 39). However only three studies demonstrated this association to be significant (17, 24, 39). Additionally, only three of 19 studies in the group which looked at fentanyl use with other substance use reported the majority of individuals being homeless (**Table 2**) (31, 32, 35). Homelessness was not reported in any of the 13 studies which examined intentional fentanyl use only (**Table 3**).

Incarceration and arrest were reported in three of the 19 studies which looked at fentanyl use with other substances (**Table 2**) (31, 32, 49). Moreover, in the group of studies that compared intentional fentanyl using cohorts with non-intentional fentanyl using cohorts, four studies reported higher rates of incarceration and arrest in cohorts who use fentanyl intentionally (**Table 4**) (24, 26, 28, 40). However, only two studies found the association to be significant (24, 28). Incarceration and arrest were not reported in the 13 studies that discussed intentional fentanyl use only (**Table 3**).

### 3.3 Overdose history

Overdose history was reported in eight of the 19 studies which did not distinguish fentanyl use and other substance use (**Table 2**) (15, 16, 21, 27, 31, 32, 36, 41). Compared to persons who did not use fentanyl in the prior six months, those that reported fentanyl use were nine times more likely to report a recent overdose following the use of any drug (16). Fentanyl injection and public injection were associated with an increased likelihood of non-fatal overdose (41). Among the group of 13 studies which looked at intentional fentanyl use only, overdose history was reported in six of the 13 studies (**Table 3**) (20, 34, 42, 50, 51, 53).

Individuals in the studies that compared intentional fentanyl using cohorts with non-intentional fentanyl using cohorts showed that individuals who use fentanyl intentionally experienced more overdoses (reported in seven out of 9 studies) (17, 23, 24, 26, 28, 39, 45). Among people who used heroin or prescription opioids from Baltimore, Boston, and Providence, fentanyl preference was associated with overdose more than a year ago (28). This is in contrast to a study by Chandra et al., 2021 where a cross-sectional survey found that those who purposefully used fentanyl any time in the past were significantly more likely to have experienced an overdose in the past 12 months (17). This finding is also supported by a study on the Australian Needle Syringe Program Survey (45). In British Columbia, Canada, even within the last 6 months, there were higher levels of non-fatal overdose in the last 6 months reported in individuals who intentionally use fentanyl compared to those who do not (39).

### 3.4 Polysubstance use

By nature of the categorization used in this review, in the group of studies that looked at fentanyl use with other substance use but where fentanyl use data were not distinguishable from other substances, it is implied that these studies included individuals who used other substances (**Table 2**). Among the group of studies that looked at intentional fentanyl use only, polysubstance use was common among participants as it was reported in nine of the 13 studies (**Table 3**) (22, 34, 38, 42, 43, 47, 48, 51, 53). This is supported by the group of studies comparing intentional fentanyl using cohorts with non-intentional fentanyl, substance using cohorts, where individuals who intentionally use fentanyl are more likely to report polysubstance use, including cocaine, heroin, and methamphetamine use (reported in eight of 9 studies) (14, 17, 23, 24, 26, 28, 39, 40). In one study, young adults who reported non-medical fentanyl use were associated with regular heroin and cocaine use, diverted pharmaceutical fentanyl use in the prior six months, regular injection drug use and prior overdose, when compared to individuals that reported non-intentional fentanyl-contaminated heroin use (24). Similarly, a population estimation study reported individuals who prefer fentanyl to have recently smoked or injected heroin and more likely to report recent injection of speedball and cocaine (26).

### 3.5 Reasons for fentanyl use

Among participants from studies on fentanyl with other substance use, five out of 19 studies reported motivations for fentanyl use (13, 27, 33, 37, 44). Motivations included seeking out fentanyl due to their high tolerance levels, (13, 27, 37) higher potency, (13, 27, 33, 37) delaying the onset of withdrawal, (27) and intense rush and feelings of euphoria (13, 44). Among the studies which looked at intentional fentanyl use only, motivations for fentanyl use were reported in six of the 13 studies (**Table 3**) (22, 38, 47, 48, 50, 51). Motivations included relieving stress/anxiety and pain (22, 38, 51). Among studies that compared intentional fentanyl using cohorts with non-intentional fentanyl substance using cohorts (**Table 4**), only one study described the motivations for fentanyl use among the sample: among participants who reported intentional fentanyl-contaminated heroin use, the majority (59%) reported that it provided a better high (24).

### 3.6 Usage patterns

Among the 41 studies included in this review, injection as a route of administration was preferred or common in more than half of the studies. This was reported in 11 of 19 studies which did not distinguish fentanyl use and other substance use. (13, 18, 21, 27, 30–32, 35, 46, 49, 52) Similarly, this was reported in four of 13 studies which looked at intentional fentanyl use only, (22, 34, 38, 53) and seven of the nine studies which differentiated intentional fentanyl using cohorts and non-intentional fentanyl use cohorts. (17, 23, 26, 28, 39, 40, 45) Fentanyl preference was also associated with documented daily illicit drug use, (28) and injection in a public location in the last month, (45) as well as daily injection use. (16)

### 3.7 Medical comorbidities

Independent correlates of any purposeful fentanyl use included moderate/severe depression. (17) In Macmadu et al., 2017, the group of individuals with intentional fentanyl-contaminated heroin use also had a higher proportion of ever testing positive for HCV and having a mental health diagnosis. (24) Among the studies which looked at intentional fentanyl use only, only 4 had reported concurrent disorders among participants: depression, (51) mental problems, (43) lifetime history of mental illness diagnosis, (22) and depression with psychosis. (34)

### 3.8 Study quality

Out of the 41 papers included in this systematic review, 19 were cross-sectional studies; three were case-control, cohort, or qualitative studies. Most of the cross-sectional studies were of good methodological quality (a score of 6 or above out of 10). Six were of moderate quality (a score of 5) due to the lack of comparability based on the study design (Supplementary material). All the 12 qualitative studies were of good quality, the only flaw being not considering the relationship between the researcher and the participants for all studies except for one. This systematic review includes 11 case reports, of which nine were of good methodological quality, and two were of low quality. One flaw they all had in common was not including patients’ perspectives or experiences. However, this was not possible for some of the papers as the subjects were deceased (**Supplementary material**).

## 4 Discussion

This systematic review found demographic indicators that were associated with fentanyl use. These include identifying as White, male, and young. Individuals who report intentional use of fentanyl also have higher likelihood of risky substance use behaviours and patterns, such as injection as their preferred route of administration, use of multiple substances, recent overdose history, daily substance use, and use of substances in public spaces. This group was also associated with socioeconomic risks such as homelessness, higher rates of unemployment, and incarceration. The scaling-up of interventions to effectively address such social and structural factors is direly needed to improve the health and well-being of individuals with fentanyl use.

Health care systems currently struggle with adapting treatment strategies to individuals with fentanyl use and severe opioid use disorder (OUD). Some novel approaches to opioid agonist treatment (OAT) have emerged in recent years, but have not seen appropriate and sustainable implementation, despite the need for it. For instance, Health Canada in 2019 approved injectable diacetylmorphine and hydromorphone for treatment of severe OUD in adults (injectable opioid agonist treatment: iOAT), (54) but the number of patients receiving iOAT is still low (149 diacetylmorphine and 28 hydromorphone clients in British Columbia in November 2022) (55). High doses of buprenorphine have been found to be effective in patients who use fentanyl in some studies due to its high potency and affinity for μ-opioid receptors (27, 56). However, the lipophilicity of fentanyl leads to its accumulation in peripheral tissues, resulting in an increased risk of precipitated withdrawal and difficulty with the buprenorphine induction process (57). One such innovation may be the use of low-dose buprenorphine inductions, which has been reported only in case series but has been successfully utilized to avoid precipitated withdrawal among fentanyl-using patients (14, 58). As there is overall limited experience with OAT approaches to suit fentanyl-using individuals, further timely research is needed to explore alternative treatment strategies, which include high-dose methadone and slow-release oral morphine protocols and fentanyl iOAT (59–62).

Current guidelines recommend the use of methadone, buprenorphine, and non-opioids for managing opioid withdrawal, however, these medications can often be insufficient in alleviating withdrawal among patient using fentanyl (63–65). Patients with undertreated withdrawal may use their illicit substances and self-discharge against medical advice, which are strongly associated with adverse outcomes and mortality (66–68). Some physicians have employed the use of short-acting opioids, like IV hydromorphone and fentanyl, to support patients to stay in hospital and initiate them on OAT (60, 65). Although these approaches have been successful, they have not yet been formally recognized as alternatives for withdrawal management in hospitalized patients. Further research is needed to determine the efficacy for these strategies.

This systematic review has several limitations. Of note are the heterogeneity of the included studies. In particular, it was important for this study to identify and focus on the intentionality of fentanyl use. Therefore, other variability was accepted in the inclusion criteria. In order to present data as granular as possible, studies where fentanyl use specifically was separated from other substances were grouped separately from studies where fentanyl use was included but not separable from the use of other substances. In addition, due to the novelty of this paper, and its focus on qualitative outcomes and breadth of data, it was difficult to screen for sufficient homogeneity to allow for a meta-analysis. Finally, this review specifically reported on intentional non-medical fentanyl use among PWUD, as opposed to among people with OUD, in order to increase the breadth of studies included and the generalizability of the findings.

## 5 Conclusion

The growing tendency to use fentanyl as drug of choice is extremely concerning. Our review has found that individuals who intentionally use fentanyl have severe substance use patterns, precarious living situations, and extensive overdose histories. With the street supply of opioids increasing in toxicity and an increasing number of individuals intentionally seeking fentanyl, more effective withdrawal management and OAT approaches must be developed. This paper calls for healthcare providers, researchers, and government advocates to develop alternative approaches for OUD and put in place policies allowing increased availability for fentanyl-based treatment options based on further research, which will result in a paradigm shift in the system of care.

## Data availability statement

The original contributions presented in the study are included in the article/**Supplementary Materials**, further inquiries can be directed to the corresponding author.

## Author contributions

VT: Conceptualization, Data curation, Formal analysis, Methodology, Project administration, Supervision, Writing – original draft, Writing – review & editing. JW: Data curation, Formal Analysis, Methodology, Writing – original draft, Writing – review & editing. JWe: Data curation, Formal analysis, Methodology, Writing – original draft, Writing – review & editing. NR: Data curation, Formal analysis, Writing – review & editing. HF: Data curation, Formal analysis, Writing – review & editing. MN: Writing – review & editing. VL: Writing – review & editing. NM: Writing – review & editing. PA: Writing – review & editing. KJ: Writing – review & editing. RK: Conceptualization, Resources, Supervision, Writing – review & editing.
