# Supplementary material for: Systematic review on intentional non-medical fentanyl use among people who use drugs
Source: Front Psychiatry. 2024 Feb 13;15:1347678. doi: 10.3389/fpsyt.2024.1347678 (PMC10896833; doi:10.3389/fpsyt.2024.1347678)
Supplement: Supplementary file 1 [file DataSheet_1.docx]

**Table S1a. Search strategy for Ovid MEDLINE (-May 2021)**

| Row | Search term | Results |
| --- | --- | --- |
| 1 | Substance-related disorders/ or drug overdose/ or opiate overdose/ or narcotic-related disorders/ or opioid-related disorders/ or heroin dependence/ or substance abuse, intravenous/ or substance abuse, oral/ | 142100 |
| 2 | Illicit Drugs/ | 11547 |
| 3 | Illicit drug*.mp. | 20534 |
| 4 | Substance-related disorder*.mp. | 98327 |
| 5 | Drug overdos*.mp. | 13725 |
| 6 | Opiate overdos*.mp. | 290 |
| 7 | Narcotic-related disorder*.mp. | 30 |
| 8 | Opioid-related disorder*.mp. | 16654 |
| 9 | Heroin depend*.mp. | 9536 |
| 10 | Substance abus*.mp. | 55639 |
| 11 | Intravenous use*.mp. | 1241 |
| 12 | 1 or 2 or 3 or 4 or 5 or 6 or 7 or 8 or 9 or 10 or 11 | 174353 |
| 13 | Fentanyl/or alfentanil/ or sufentanil | 16221 |
| 14 | Fentanyl.mp. or alfentanil.mp. or sufentanil.mp. | 23497 |
| 15 | 13 or 14 | 25712 |
| 16 | 12 and 15 | 1248 |
| 17 | Limit 16 to (English language and humans and (*all adult (19 plus years)* or adolescent (13 to 18 years)*)) | 500 |

**Table S1b. Search strategy for Web of science (-May 2021)**

| Row | Search term |
| --- | --- |
| 1 | (TS=(substance-related disorder* OR drug overdose OR opiate overdose OR narcotic-related disorder* OR opioid-related disorder* OR heroin dependence OR  substance abuse OR intravenous) ) *AND* LANGUAGE: (English) *AND* DOCUMENT TYPES: (Article)  *Indexes=SCI-EXPANDED, SSCI, A&HCI, CPCI-S, CPCI-SSH, ESCI Timespan=Last 5 years* |
| 2 | (TS=(fentanyl OR alfentanil OR sufentanil) ) *AND* LANGUAGE: (English) *AND* DOCUMENT TYPES: (Article)  *Indexes=SCI-EXPANDED, SSCI, A&HCI, CPCI-S, CPCI-SSH, ESCI Timespan=Last 5 years* |
| 3 | Combine 1 AND 2 |
| Results | 1503 |

**Table S1c. Search strategy for PsychINFO (-May 2021)**

| Row | Search term |
| --- | --- |
| 1 | substance-related disorder* OR drug overdose OR opiate overdose OR narcotic-related disorder* OR opioid-related disorder* OR heroin dependence OR substance abuse OR intravenous [ALL TEXT] |
| 2 | fentanyl OR alfentanil OR sufentanil [ALL TEXT] |
| 3 | Combine 1 AND 2 |
|  | Narrow by human results only, years 2010-2021, English only, and articles only |
| Results | 278, 89 after duplication screening |

**Table S1d. Search strategy for EMBASE (-May 2021)**

| Row | Search term | Results |
| --- | --- | --- |
| 1 | Substance-related disorders/ or drug overdose/ or opiate overdose/ or narcotic-related disorders/ or opioid-related disorders/ or heroin dependence/ or substance abuse, intravenous/ or substance abuse, oral/ | 182418 |
| 2 | Illicit Drugs/ | 16321 |
| 3 | Illicit drug*.mp. | 25602 |
| 4 | Substance-related disorder*.mp. | 4949 |
| 5 | Drug overdos*.mp. | 34191 |
| 6 | Opiate overdos*.mp. | 493 |
| 7 | Narcotic-related disorder*.mp. | 2 |
| 8 | Opioid-related disorder*.mp. | 1220 |
| 9 | Heroin depend*.mp. | 10244 |
| 10 | Substance abus*.mp. | 74673 |
| 11 | Intravenous use*.mp. | 1393 |
| 12 | 1 or 2 or 3 or 4 or 5 or 6 or 7 or 8 or 9 or 10 or 11 | 220792 |
| 13 | Fentanyl/or alfentanil/ or sufentanil | 81821 |
| 14 | Fentanyl.mp. or alfentanil.mp. or sufentanil.mp. | 87053 |
| 15 | 13 or 14 | 87053 |
| 16 | 12 and 15 | 4114 |
| 17 | Limit 16 to (English language and humans and (*all adult (18-64 years)* or adolescent (13 to 17 years)*)) | 1563 |

**Table S2. Additional information on included studies (N=41)**

|  | **Title** | **Objective of study** | **Sample size (N)** | **Substances of use (types and % if available)** | **Sample distribution (all fentanyl or mixed, what %)** | **How did study determine intentional fentanyl use** | **Is it possible to extract data separately for intentional fentanyl users?** | **Recruitment method** | **City, country** | **Timelines** | **Methodology of study** | **Method of data analysis** | |
| --- | --- | --- | --- | --- | --- | --- | --- | --- | --- | --- | --- | --- | --- |
| **Studies Involving Intentional Fentanyl Use That is Undistinguishable from Other Substance Use (N=19)** | | | | | | | | | | | | |  |
| Amlani et al (2015)^43^ | Why the FUSS (Fentanyl Urine Screen Study)? A cross-sectional survey to characterize an emerging threat to people who use drugs in British Columbia, Canada. | Assess the prevalence and characteristics of fentanyl use among clients accessing harm reduction services in BC. | 242 | Crystal methamphetamine (58 %), heroin (52 %), fentanyl (13%), Methadone (30%), Morphine (40%), Dilaudid (23%), Oxycodone (10%), Benzodiazepines (20%), Stimulants NOS (10%), Heroin (52%), Cocaine powder (27%), Crack (32%), Marijuana (23%) | Individuals who reported using fentanyl (13%) | Self-reported intentional use | No | Participants attending HR services across BC were invited to complete an anonymous questionnaire and provide a urine sample to test for fentanyl. | British Columbia, Canada | Feb - March 2015 | Cross-sectional design linking surveys of demographics and substance usage patterns with fentanyl urine tests. | Descriptive and bivariate analysis, multivariate statistical methods | |
| Bach et al (2020)^20^ | Prevalence of fentanyl exposure and knowledge regarding the risk of its use among emergency department patients with active opioid use history at an urban medical center in Baltimore, Maryland | The prevalence of fentanyl exposure, patients’ knowledge regarding potency and risk of overdose death from fentanyl and intentional purchase of fentanyl. | 165 (survey), 129  9urine samples for fentanyl testing) | Study cohort: Ethanol (43.6%), Marijuana (45.5%), Cocaine (57.6%), Benzodiazepines (20.6%), SCRAs (4.8%). Fentanyl positive: Ethanol (44.2%), Marijuana (42.3%), Cocaine (55.7%), Benzodiazepines (22.1%), SCRAs (1%) | Fentanyl positive (80.6%) | Survey | No. | Identified by research assistants through tracking the triage screening of ED patients for substance use an urban community hospital | Baltimore, Maryland, USA | May - July 2018 | Cross-sectional study-  urine analysis and survey | Urine analysis | |
| Balsamo et al (2021)^60^ | Patterns of routes of administration and drug tampering for nonmedical opioid consumption: Data mining and content analysis of reddit discussions | To find a large cohort of Reddit users interested in discussing the use of opioids, trace the temporal evolution of their interest, and extensively characterize patterns of the nonmedical consumption of opioids, with a focus on routes of administration and drug tampering. | 86,445 | N/A | Mixed (no % available) | Information provided on Reddit comments | No | Publicly available Reddit data set | Online - Reddit | 2014- 2018 | Semi Automatic information retrieval algorithm Reddit | Statistical modeling | |
| Bardwell et al (2021)^62^ | People need them or else they’re going to take fentanyl and die”: A qualitative study examining the ‘problem’ of prescription opioid diversion during an overdose epidemic | Highlight the intricate means by which POs (prescription opioids) are acquired and diverted and how environmental contexts frame how participants negotiated risk and rationalized diversion. | 21 | Opioid prescribed:  methadone (28.6%), hydromorphone (23.8%),, acetaminophen/codeine (14.3%), morphine (14.3%), acetaminophen/oxycodone (9.5%), fentanyl (transdermal) (4.8%), buprenorphine/naloxone (4.8%)  Drug preference:  heroin (38.1%), fentanyl (33.4%), prescription opioids (23.8%), crystal methamphetamine (14.3%), cocaine (14.3%), alcohol (9.5%), speedball (4.8%), cannabis (4.8%) | Preferred fentanyl (33.4%) | interview | No | Recruited from two cohorts studies: ACCESS and VIDUS | Vancouver, Canada | Dec 2019- March 2020 | Qualitative semi-structured interviews | Thematic coding | |
| Buresh et al (2019)^21^ | Recent fentanyl use among people who inject drugs: Results from a rapid assessment in Baltimore, Maryland. | Assess knowledge, attitudes and practices related to fentanyl use in a community-based cohort of former and current PWID | 994 | Cocaine (42%), opioid (41%), marijuana (18%), Alcohol (48%) | Mixed; ever used fentanyl (28%) | Self-reported intentional use | Yes | Local syringe services providers, HIV treatment providers, word-of-mouth, and community events | Baltimore, Maryland | Nov 2017- June 2018 | Cross-sectional study quantitative survey | Standard descriptive statistic, multivariable analysis | |
| Ciccarone et al (2017)^22^ | Heroin uncertainties: Exploring users' perceptions of fentanyl-adulterated and -substituted 'heroin'. | To understand the perceptions and experiences of people who inject drugs sold as ‘heroin’ and to observe the drugs and their use. | 38 | Persons who use heroin | No % provided but they describe types of opioids sold: heroin (alone), fentanyl (alone), and heroin– fentanyl combinations. | Qualitative interview | No | Recruited during their daily activities including living at a homeless encampment, sitting around outside, meeting up with friends, as well as visiting harm reduction services. Most were introduced by harm reduction workers, but some snowball sampling was also carried out. | Northeast Massachusetts and Nashua, New Hampshire | June 2016 | Qualitative rapid ethnographic study and semi-structured interview | Analytic memos, inductive analysis | |
| Daniulaityte et al (2019)^23^ | Street fentanyl use: Experiences, preferences, and concordance between self-reports and urine toxicology. | How PWUD tell fentanyl apart from heroin | 60 | NFP (73.3%), Heroin (58.3%), Alcohol (41.7%), Marijuana (66.7%), Cocaine (80%), Methamphetamine (43.3%), Diverted Pain Pills (43.3%), Diverted Benzodiazepines (55%), Diverted Gabapentin (26.7%), diverted Buprenorphine (43.3%) | Street Fentanyl ONLY, NO Heroin (23.3%), Street Fentanyl AND Heroin (48.3%), Heroin ONLY, NO Street Fentanyl (10%), NO Heroin, NO Street Fentanyl (11.7%) | Self-reported heroin/NPF use | No | Craigslist ads, flyers, referrals | Dayton, Ohio, US | May 2017-Jan 2018 | Semi structured interviews and urine drug screen | Quantitative data via SPSS using descriptive statistics, PPV, NPV, Cohen’s kappa computed | |
| Gryczynski, et al (2019)^24^ | Fentanyl exposure and preferences among individuals starting treatment for opioid use disorder | To examine correlates of fentanyl exposure among admissions to a community OUD treatment program, and to explore patient experiences and preferences regarding fentanyl. | 1,174 (review records); 114 (responses to anonymous survey) | Positive for fentanyl (39.35%) Preferred opioid of choice: Heroin alone (40.4%) Rx opioids (11.4%) Multiple, not including fentanyl (3.5%) Fentanyl alone (5.3%) Fentanyl and heroin mix (33.3%) Multiple, including fentanyl (6.1%) Fentanyl (alone or mix): 44.7% | Having knowingly taken fentanyl that was sold on the street (56.1%), knowing people for whom fentanyl was a preferred drug of choice (65.8%). With respect to personal preferences, only 5.3% reported fentanyl alone as their preferred drug of choice (5.3%), preference for “fentanyl and heroin mix” (33.3%) | Survey (only descriptive results due to limited sample size) | No | Convenience sampling of outpatients for interview and admission intake for UDS | Baltimore, Maryland, US | Electronic health records for treatment admissions during a 7-month period in 2018. Brief one-page anonymous survey in September 2018. | Review of treatment records and anonymous survey | Descriptive, Pearson chi-square tests of independence for categorical variables and independent samples t-tests for continuous variables, logistic regression model | |
| Kenney et al (2018)^25^ | Expected and actual fentanyl exposure among persons seeking opioid withdrawal management | Compared beliefs, behaviors, and risk perceptions related to fentanyl with actual toxicology reports among people who used opioids. | 231 | Heroin in past month (92.2%), fentanyl intentionally (70.4%), fentanyl unintentionally (15.2%) | Fentanyl intentionally (70.4%), fentanyl unintentionally (15.2%) | Interview | No | Persons seeking inpatient opioid withdrawal management were asked to participate in a survey research study | Fall River, Massachusetts | April - Sept 2017, | Survey | Quantitative, descriptive, inferential and urine statistics | |
| Krause et al (2017)^53^ | High Concomitant Misuse of Fentanyl in Subjects on Opioid Maintenance Treatment | To investigate the occurrence of concomitant fentanyl misuse in opioid dependent patients on opioid maintenance treatment and to study the correlates of fentanyl misuse. | 960 (UDS) and 401 (questionnaire) | From urine analysis of OAT drugs: methadone (73.5%), buprenorphine (17.5%), diamorphine (9%)  Total fentanyl misuse detected by UDS (6.8%) | Urine samples analysis revealed that positive for fentanyl (6.8%), stated they had taken fentanyl as a concomitant drug (37.9%), | Questionnaire | No | recruited from outpatient clinics | Munich, Germany | 2008 to 2012 | Cross sectional- Urine analysis and questionnaire | Quantitative | |
| McLean et al (2019)^26^ | "You Never Know What You're Getting": Opioid Users' Perceptions of Fentanyl in Southwest Pennsylvania | Discussing user perceptions of fentanyl as both danger and opportunity, the risk environment created by individual perceptions and particular geographic contexts | 125 surveys, 30 interviews (30/125) | Opioids (no% available) | Mixed with heroin (intentional or unintentional) (100%) | Interviews | No | Targeted sampling, advertised the survey in local drug treatment clinics and public locations | Allegheny, Fayette, Greene, Washington | July 2017 - July 2018 | Qualitative interview and surveys | NVivo, Pearson’s chi-squared | |
| Moeller et al (2020)^50^ | ``Shop Until You Drop{''}: Valuing Fentanyl Analogs on a Swedish Internet Forum | Examine user perceptions of fentanyl analogs | 24 threads with 8761 posts on Flashback.org | Fentanyl related threads | Fentanyl related threads | Some of the users purchased fentanyl analogs wittingly | N/A | [Online through flashback.org](http://flashback.org/) | Sweden | Sept 13, 2012-July 26, 2019 | Thematic analysis of a public internet forum Flashback | Thematic analysis | |
| Nolte et al (2020)^27^ | Opioid initiation and injection transition in rural northern New England: A mixed-methods approach | Assess the risk environment for opioid initiation and transition to injection among people who use drugs (PWUD) in rural northern New England | Audio computer-assisted self-interview surveys (n = 589),  shared personal narratives through in-depth interviews (n = 22) | From the ACASI: heroin as drug of choice (60 %) followed by cocaine/ crack (16%), opiate pain killers (7%), buprenorphine (4%), street fentanyl (4%), and methamphetamine (4%). Among participants currently injecting drugs (n = 453): drugs most injected in the past 30 days: heroin (87%), street fentanyl (59%), cocaine/crack (50%), speedball (heroin and cocaine) or screwball (heroin and methamphetamine) (33%), opiate pain killers (26%), methamphetamine (25%), and buprenorphine (25%) | Mixed | Interviews | No | Recruitment was conducted at 11 study sites selected in consultation with local public health officials and service providers. Field staff recruited 51 seeds through street outreach and at harm reduction agencies, and 538 participants were referred to the study through respondent driven sampling methods. | Rural Northern New England, Northeastern United States, | May 2018 – Oct 2019 | Interview and survey | Descriptive analysis and thematic analysis | |
| O'Rourke et al (2019)^28^ | Acceptability of safe drug consumption spaces among people who inject drugs in rural West Virginia. | Examines hypothetical SCS acceptability among rural sample of PWD in West Virginia due to disproportionate impact of opioid crisis in rural communities where there is no SCS | 373 | Injected drugs: cocaine (35.4%), heroin (82.0%), speedball (37.8%), crystal methamphetamine (71.0%), fentanyl (56.3%), buprenorphine or suboxone (29.8%), and painkillers (21.7%) | Prefer fentanyl (30.1%) | Cross-sectional survey | No | Recruited PWID from a syringe services program and in community locations where PWID were known to congregate | Cabell County, West Virginia USA | June - July 2018 | Cross sectional survey with statistical analysis | Chi-square and t tests | |
| Park et al (2019)^29^ | Willingness to Use Safe Consumption Spaces among Opioid Users at High Risk of Fentanyl Overdose in Baltimore, Providence, and Boston | Identify the factors associated with willingness to use a SCS among injectors and nanoinjectors | 326 | Heroin injected (61%), heroin smoked/snorted (40.8%), Injected cocaine (33.7%), Crack cocaine (73%), powdered cocaine (25.2%), Heroin and cocaine "speedball" (40.2%), non-medical prescription opioid pill use (36.2), Methamphetamine (11.4%), non-medical benzodiazepine use (42.9%) | Mixed Opioids (heroin, fentanyl, and non-medical opioid pill use) | Survey, 26% reported a preference for drugs with fentanyl | No | Targeted sampling based on geospatial mapping of data from the Baltimore City Police Department, syringe service programs (SSP) and harm reduction services. | Baltimore, Maryland; Boston, Massachusetts; and Providence, Rhode Island | June and Oct 2017 | Survey, interview | Pearson’s chi-squared tests, bivariate and multivariate logistic regression analyses, stratified analysis, | |
| Silverstein et al (2019)^30^ | "Everything is not right anymore": Buprenorphine experiences in an era of illicit fentanyl. | Explore local perceptions of the presence of non-pharmaceutical fentanyl and how this impacts practices of non-prescribed buprenorphine use among PWUIO | 63 | Ever knowingly used non-pharmaceutical Fentanyl (93.7%)  ever used heroin (98.4%)  ever used non-prescribed buprenorphine (87.3%) | Heavy heroin/fentanyl, low NBP (15.3%); More Use of Formal Treatment and Low NPB Use (16.2%); Intense NPB Use, Less Formal Treatment (37.1%) | N/A | No | Community outreach | Dayton, US | May 2017-June 2019 | Qualitative interviews | NVivo, thematic coding | |
| Uuskula et al (2017)^56^ | HIV prevalence and gender differences among new injection-drug-users in Tallinn, Estonia: A persisting problem in a stable high prevalence epidemic. | To examine HIV prevalence and gender differences in HIV prevalence and risk behavior among new injection drug users in Tallinn, Estonia. | 110 | 63% reported injecting mainly fentanyl, 34% injecting mainly amphetamine | Reported injecting mainly fentanyl (63%) | Interviewer-administered questionnaire | No | Respondent-driven sampling | Tallinn, Estonia | Combined data from 3 studies, conducted biannually from 2009 to 2013 | Cross-sectional and observational | Quantitative analysis | |
| Wallace et al (2019)^45^ | Factors Associated with Nonfatal Overdose During a Public Health Emergency. | Examine factors associated with recent non-fatal overdose during a time of unprecedented rates of overdose and increasing involvement of fentanyl and fentanyl derivatives in overdose deaths | 187 | Heroin, fentanyl, cocaine, crystal methamphetamine, alcohol (no % available) | Daily fentanyl use (30.4%) and 26 (46.4%), fentanyl at least once but < daily in the previous 6 months (46.4%). | Self-reported intentional use | No | Convenience sampling from sites distributing clean injecting supplies | Victoria, Canada | June - Sept 2016 | Cross-sectional survey | Bivariable and multivariable logistic regression analysis | |
| Weicker et al (2020)^31^ | Agency in the fentanyl era: Exploring the utility of fentanyl test strips in an opaque drug market | To explore how people who use fentanyl and health‐care providers engaged in and responded to overdose risk communication interactions. | 20 | Fentanyl, heroin, and other opioids (no % available) | N/A | Interviews | No | Street outreach in targeted locations informed by Baltimore drug arrest data | Baltimore US | Oct 2018 – Dec 2019 | Qualitative interviews | MAXDQA via priori and inductive codes | |
| **Studies Involving Intentional Fentanyl Use Only (N=13)** | | | | | | | | | | | | |  |
| Eiden et al (2017)^55^ | High opioids tolerance due to transmucosal fentanyl abuse. | To illustrate a case of high opioids tolerance due to abuse of transmucosal fentanyl in a patient without history of drug abuse | 1 | He reported the use of transmucosal fentanyl (TMF) medication (Pecfent©, fentanyl pectin nasal spray) since October 2015. He consumed the TMF prescribed to his wife for terminal colorectal cancer.  No history of prior drug use | N/A | Self-reported intentional use | N/A | Case report, patient admitted to emergency department | France | May 2016 | Case report | Descriptive | |
| Firestone et al (2009)^46^ | Fentanyl use among street drug users in Toronto, Canada: behavioural dynamics and public health implications. | Explore practices and risk dynamics associated with Fentanyl abuse as well as considering public health implications. | 25 | History of illicit PO fentanyl injection within at least the past 3 months (56%) | Reported history of illicit PO fentanyl injection within at least the past 3 months (56%) | Qualitative interviews | No | Local street drug user populations with the help of community-based service provider and peer contacts | Toronto, Ontario, Canada | March – June 2007 | Exploratory (interview-based) qualitative study | N/A | |
| Gecici et al (2010)^58^ | Fentanyl dependence caused by the non-medical use: A case report | Report fentanyl dependency in a patient who had started to use fentanyl without any medical indication. | 1 | Fentanyl | Fentanyl (100%) | Self-reported intentional use | Yes | Inpatient hospital admission | Turkey | 2010 | Case report | Case report | |
| Guerrieri et al (2017)^51^ | Acrylfentanyl: Another new psychoactive drug with fatal consequences. | Documentation of acrylfentanyl cases | 40 | *Numbers indicate # of cases*: Butyrfentanyl (1) Acetylfentanyl (34) 4-Fluoro-butyrfentanyl (1) 4-Metoxibutyrfentanyl (1) Furanylfentanyl (10) Acrylfentanyl (43) 4F-isobutyrfentanyl (14) 4Cl-isobutyrfentanyl (3) Tetrahydrofuranfentanyl (5) | Fentanyl (100%) | Witnesses, 6 cases were possibly suicide attempts; other cases were considered accidental overdoses | No | Report of a series of forty fatal intoxications occurred in Sweden | Sweden | April-Oct 2016 | Femoral blood analysis and case studies | Analyst1 1.6.2 software | |
| Gunn et al (2021)^32^ | Age-based Preferences for Risk Communication in the Fentanyl Era: “A lot of people keep seeing other people die and that’s not enough for them.” | To explore how people who use fentanyl and health‐care providers engaged in and responded to overdose risk communication interactions, and how they might vary by age. | 21 | Fentanyl (no % available) | Fentanyl (no % available) | Interviews | N/A | Flyers and staff referrals at local syringe service programs, community outreach services, and primary care practices. | Boston, MA, United States. | May-Nov 2018 | Qualitative interviews | Nvivo and a grounded content analysis (deductive and inductive thematic development approaches) | |
| Kilwein et al (2018)^33^ | A descriptive examination of nonmedical fentanyl use in the United States: Characteristics of use, motives, and consequences. | descriptive examination of non medical Fentanyl use and motivation | 122 | N/A | fentanyl (100%), unintentional Fentanyl use (12.3%) | Questionnaire (descriptive survey) | no | targeting various geographic locations with online postings, through the volunteers’ section of Craigslist, Facebook and Drug Forums | 34 US states | Feb 2016 – April 2017 | Descriptive study, fentanyl questionnaire | N/A | |
| Kimergard et al (2018)^54^ | Characteristics of opioid-maintained clients smoking fentanyl patches: The importance of confirmatory drug analysis illustrated by a case series and mini-review. | To describe a case series of clients seeking drug addiction treatment for fentanyl patch smoking in Denmark | 14 | Fentanyl (100%) | Fentanyl (100%) | Medical history during intake as a patient | N/A | Outpatients who sought treatment for fentanyl smoking from an addiction service | Southern Denmark | Aug- Dec 2015 | Retrospective case review | N/A | |
| Lyttle et al (2012)^57^ | Transdermal fentanyl in deliberate overdose in pediatrics | Case report | 1 | Transdermal fentanyl (100%) | Transdermal fentanyl (100%) | Applied 5 patches to end her life | Yes | N/A | Bristol, UK | N/A | Case report | Case report | |
| Marquardt et al (2008)^34^ | Inhalation abuse of fentanyl patch. | Case report describing a man becoming intoxicated from fentanyl by heating and inhaling contents from a fentanyl patch | 1 | n = 1, fentanyl patch use | N/A | observed by paramedics | No | Man seen by paramedics | Sacramento, California, United States | 1994 | Case report | Descriptive | |
| Mrvos et al (2012)^35^ | Whole fentanyl patch ingestion: a multi-center case series | characterize whole fentanyl patch ingestion to develop a clinical guideline for management | 76 | Fentanyl patch (100%) | Fentanyl patch (100%) | Reported patients who ingested intact fentanyl patches with a history of whole patch ingestion as reported to three Regional Poison Information Centers (RPIC) | Yes | Three RPIC medical record databases | Pittsburgh, USA | 2000 – 2008. | Retrospective case review | Descriptive statistics | |
| Reeves et al (2002)^59^ | Fatal intravenous misuse of transdermal fentanyl. | We present the first documented fatality after intravenous injection of the contents of a transdermal fentanyl patch. | 1 | Fentanyl transdermal patch | Fentanyl (100%) | Injected content of transdermal patch (It was later confirmed she had shared and injected intravenously the contents of a transdermal fentanyl patch found at the scene with an individual who recovered) | No, only included patients with intentional fentanyl use | Inpatient hospital admission | N/A | 2002 | Case report | N/A | |
| Tharp et al (2004)^36^ | Fatal intravenous fentanyl abuse: four cases involving extraction of fentanyl from transdermal patches. | Present 4 cases occurring in which the fatal dose of fentanyl was extracted from transdermal patches and injected intravenously | 4 | Fentanyl transdermal Patch and injected (100%) | Fentanyl (100%) | Transdermal patch use and injected (1 suicide, other 3 police found syringes, needles fentanyl patches) | No, only patients included had fentanyl use | Post-mortem analysis | North Carolina, USA | January 1997 – July 2001 | Post-mortem analysis | N/A | |
| Woodall et al (2008)^47^ | Oral Abuse of Fentanyl Patches (Duragesic): Seven Case Reports | Case studies providing information regarding the oral transmucosal abuse of transdermal fentanyl patches and the wide range of fentanyl concentrations that can arise in deaths following this type of administration | 7 | Fentanyl patches (Duragesic) | fentanyl patches (Duragesic) | Witness reports and the finding of fentanyl patches in the oral cavity or pharynx during autopsy | No, all were using fentanyl | Identified via a retrospective analysis of fentanyl-related deaths | Ontario, Canada | Jan 2002 – Dec 2004 | Post-mortem autopsy, blood fentanyl concentrations and additional toxicological findings | Toxicological analyses | |
| **Intentional Fentanyl Using Subpopulations Among Studies Comparing Intentional Fentanyl with Non-intentional Fentanyl (N=9)** | | | | | | | | | | | | |  |
| Antoine et al (2021)^37^ | Method for Successfully Inducting Individuals Who Use Illicit Fentanyl Onto Buprenorphine/Naloxone | Case series describes buprenorphine/naloxone inductions of 4 individuals who tested positive for fentanyl | 4 | SUD history: Using mixture of heroin and fentanyl (100%)- previous 30 days: heroin/fentanyl capsules (3 cases), fentanyl capsules (1 case) | 3 of the participants: using mixture of heroin and fentanyl, 1 using fentanyl capsules in the previous 30 days - see Table 1 | Self-reported intentional use | Yes | Participants were part of RCT of a sleep medication during opioid tapering | Not clear what city, but all authors from Maryland; United States | 1 day in 2021 | Case series | Descriptive analysis | |
| Chandra et al (2021)^38^ | Purposeful Fentanyl Use and Associated Factors among Opioid-Dependent People Who Inject Drugs | Characterize purposeful fentanyl use among PWID | 104 | Any fentanyl use (43.2%), Recent fentanyl use (22.1%), Heroin (85.6%), Cocaine (78.9%) | Any fentanyl use (43.2%), Recent fentanyl use (22.1%), Heroin (85.6%), Cocaine (78.9%). | Self-reported intentional use | Yes, but we don't know if people reporting no fentanyl use were exposed to it or not - UDS not done in study. | Recruited from New England’s largest addic- tion treatment setting (APT Foundation, Inc.) using clinic-based advertisements and flyers, word-of-mouth, and direct referral from counselors. | New Haven, Connecticut, US | July 2018 - October 2019 | Cross-sectional- survey using an audio computer-assisted self-interview | Multivariable logistic regressions, Bivariate analyses | |
| Geddes et al (2018)^52^ | Intravenous fentanyl use among people who inject drugs in Australia. | Analysis of PWU fentanyl in last 6 months using survey across Australia with serological testing | 2378 | Among people who had injected PO in the last 6 moths: Fentanyl (23%). Among those who recently injected fentanyl: heroin last drug injected (32%), | Fentanyl (23%) | Survey | N/A | Through the annual Australian NSP survey | All of Australia | 2014 | Cross sectional - self-administered questionnaire and antibody testing. | Bivariate analysis, logistic regression models, data analysis via STATA version 14.0 | |
| Karamouzian et al (2020)^48^ | Known fentanyl use among clients of harm reduction sites in British Columbia, Canada. | Urine analysis of reported known fentanyl use, unknown fentanyl use and no recent fentanyl use | 303 | Cannabis (50.5%), methadone (25.7%), heroin/morphine (50.8%), oxycodone (4.6%), crystal meth (62%), cocaine (19.5%), crack (21.5%), benzodiazepine (11.6%) | Reported known fentanyl use (38.7%), had unknown fentanyl use (21.7%), no recent fentanyl (39.6%) use | Self-reported intentional use in the previous three days | Yes | Recruited from harm reduction sites | British Columbia, Canada | May - August 2018 | Cross-sectional study, completed a urine sample to test for fentanyl and a survey | Bi-variable and multivariable multinomial logistic regression models, modified Poisson regression model with robust error variance. | |
| Kline et al (2021)^39^ | Opioid overdose in the age of fentanyl: Risk factor differences among subpopulations of overdose survivors | Addresses the gaps by exploring the association between overdose and fentanyl-related attitudes/behaviors in three subpopulations of overdose survivors. | 432 | Often/always mixes opioids with one or more other opioids (60%) (including those who mixed opioids with benzodiazepines (28%), with cocaine (41%), with methamphetamine (11%)) heavy alcohol use (19.7%) | Heroin seizures containing fentanyl in country of residence (51.4%) | Survey | No | Existing patients in methadone maintenance programs (67%) and acute residential detoxification programs (33%) | New Jersey, USA | October 2018 - March 2019 | cross-sectional quantitative study | Bivariate and multivariate analysis | |
| Macmadu et al (2017)^40^ | Prevalence and correlates of fentanyl-contaminated heroin exposure among young adults who use prescription opioids non-medically. | To examine risk factors for exposure to fentanyl-contaminated heroin and experiences with its use among young adult NMPO users. | 199 | known or suspected FCH exposure (11%), Heroin (25.6%), non-medical prescription opioid (47.2%), Cocaine (11.1%), non-medical benzodiazepine use (30.2%), | Known or suspected FCH exposure (11%) | Self-reported intentional use | Yes | Targeted canvassing, snowball sampling, and internet-based recruitment from online classifieds, drug information websites, and social media. | Rhode Island, United States | Jan2015 – Feb 2016 | Cross-sectional study with interviews and surveys. | Bivariable logistic regression models and descriptive statistics | |
| Mazhnaya et al (2020)^41^ | Fentanyl Preference among People Who Inject Drugs in West Virginia | To investigate factors associated with fentanyl preference among rural PWID. | 311 | All have used fentanyl in their lifetime | Total (N=311): Fentanyl (68.8%), Heroin (91.6%), Buprenorphine (27.7%), Painkillers(23.5%), Crystal metamphetamine (75.2%), speedball (44.1%), Cocaine (38.9%)  of the users who Prefer drugs containing fentanyl (N=135): Fentanyl (83.7%), Heroin (97.8%), Buprenorphine (25.2%), Painkillers(25.2%), Crystal methamphetamine (76.3%), speedball (49.6%), Cocaine (49.6%)  of the users who do not Prefer drugs containing fentanyl (N=176): Fentanyl (57.4%), Heroin (86.9%), Buprenorphine (29.6%), Painkillers(22.2%), Crystal methamphetamine (74.3%), speedball (35.2%), Cocaine (30.7%) | Survey | Yes | Purposive sampling at the harm reduction program and community | Cabell County, WV | June- July 2018. | Audio computer-assisted self-interview (ACASI) | Chi-square tests for categorical variables and Kolmogorov- Smirnov, Multivariable Poisson regression with robust variance estimation | |
| Mitra et al (2020)^49^ | Elevated prevalence of self-reported unintentional exposure to fentanyl among women who use drugs in a Canadian setting: A cross-sectional analysis | Examined differences in self-reported unintentional exposure to fentanyl between men and women who use drugs. | 578 | those with fentanyl use in last 30 days: heroine (60.9%), prescription opioid (3.6%), daily stimulant use (43.5%) | Fentanyl (100%) | Self-reported intentional use | Yes | Self-referral and community outreach | Vancouver, Canada | Dec 2016- Nov 2017 | Questionnaire | Pearson's Chi-squared test, multivariable logistic regression model, | |
| Morales et al (2019)^42^ | Preference for drugs containing fentanyl from a cross-sectional survey of people who use illicit opioids in three United States cities | Examine characteristics of individuals affected by high rates of fentanyl-related overdose deaths and address a knowledge gap regarding who prefers fentanyl and provide insight as to why they do so, | 308 | Heroin injection (67.9%), heroin smoked or snorted (46.1%), crack cocaine (70.5%), snort powdered cocaine (25.6%), cocaine injection (37.7%), speedball injection (44.8%) | Do not prefer fentanyl (73%), prefer fentanyl (27%) | Self-report via survey | Yes 'prefers fentanyl' | Targeted recruitment based on 2016 drug arrest data Boston and Providence utilized convenience sampling at harm reduction organizations | Baltimore, Maryland (8 sites), Boston, Massachusetts (1 site), and Providence, Rhode Island (3 sites). | June – Oct 2017 | Cross sectional surveys to PWUD who illicitly used heroin or prescription opioids in the prior 6 months | Pearson’s chi-square tests | |

*Abbreviations: NPF: non-pharmaceutical fentanyl, PWU: people who use, SSP: syringe service programs, HR: harm reduction, UDS: urine drug screening, HIV: human immunodeficiency virus, N/A: not applicable.*

**Table S3. Intentional fentanyl using subpopulations among studies comparing intentional fentanyl with non-intentional fentanyl (N=9)**

|  | **Intentional fentanyl using group** | **Age** | **IV use** | **Gender** | **Substance use patterns** | **Overdose** | **Racial/Ethnic** | **Socioeconomic** | **Motivation of use** |
| --- | --- | --- | --- | --- | --- | --- | --- | --- | --- |
| Antoine et al (2021)^37^ | Intentional fentanyl use (n=3) | 18-25: 33% (1/3) 26-40: 33% (1/3) 41-55: 33% (1/3) | NR | Male: 100% | Heroin/fentanyl use intranasal: 100% (3/3) Prescription opioid misuse: 33% (1/3) Cocaine use: 66% (2/3) | N/R | Caucasian: 100% (3/3) | N/R | N/R |
| Chandra et al (2021)^38^ | Purposeful fentanyl use (n=45) | Mean (SD): 37.5 (8.8) | Inject daily: 28.9% | Male: 64.4% Female: 35.6% | Heroin: 86.7% Cocaine: 88.9% Poly drug: 91.1% | Non-fatal OD in past year: 28.9% | White: 82.2% Non-white: 17.8% | High school graduate: 75.6% Income level < $10,000: 68.9% Currently married/living with partner: 28.9% Homeless in past year: 60.0% | N/R |
| Geddes et al (2018)^52^ | Recent fentanyl injection (n=193) | <30 years: 12% 30–39 years: 43% 40–49 years: 33% >49 years: 12% | Injecting initiation (years) <9: 16% 10–19: 33% 20–29: 34% >29: 16%  Daily injection: 78% Public injecting: 53% Reused syringes: 29% Used another’s used drug preparation item: 41% Used another’s used syringe: 18%  All last month | Female: 23% Male: 75%  Heterosexual: 83% Bisexual: 9% Homosexual: 2% | Fentanyl as the main opioid in last 6 months: 78% Heroin as the drug last injected: 32%  Currently in OST: 37% | Overdose in last 12 months: 37% | Indigenous Australian: 20% Non-indigenous: 77% | HCV negative: 38% HCV positive: 62% | N/A |
| Karamouzian et al (2020)^48^ | Known use (n=117) | ≥50: 22.2% 40–49: 32.1% 30–39: 52.2% 19–29: 48.33% | Preferred route of administration  injection: 55.5% non-injection: 22.8% | Men: 38.7%  Women: 38%  LGBTQ: 37.5% | Cannabis: 39.2% Methadone: 64.1%  Heroin/morphine: 61.8% Oxycodone: 85.7% Crystal meth: 55.3%  Cocaine: 57.6% Crack: 52.3% Benzodiazepine: 51.4% Polydrug: 66.4% | Experienced non-fatal overdose in the last 6 months: 51.8% | N/R | Unstable housing (current): 52.3% Paid employment: 27.2% Medium/large urban cities: 48.1% Small urban/rural communities: 22.3% | N/R |
| Kline et al (2021)^39^ | Persistent overdose subgroup* (n=40) | Mean (SD): 38.03 (11.49) | IV Injection: 72.5% | Male: 72.5% | Often/always mixes opioids with one or more other drugs: 70% Heavy alcohol use: 40% | Mean number of overdoses: 8.03 | White: 69.2% African American: 15.4% Hispanic: 15.4% | Homeless: 15% | Believes fentanyl is an important cause of overdose: 90.0% Believes fentanyl is a dangerous drug: 87.5% Prefers fentanyl to other opioids: 56.4% |
| Macmadu et al (2017)^40^ | Self-report intentional use of fentanyl-contaminated heroin in prior 6 months (n=22) | Median (IQR): 27 (25-28) | Injection drug use: 40.9% | Male: 72.7% Female: 27.3% | Heroin: 81.8% NMPO: 63.6% Cocaine: 36.4% Non-medical benzodiazepine: 59.1% Diverted pharmaceutical fentanyl: 72.7%  Daily use of FCH: 14% FCH use at least every week: 36% | Ever experienced a non-fatal overdose: 63.6% | White, non-Hispanic: 95.5% Other: 4.5% | Education beyond high school: 45.5% Ever detained in jail: 59.1% Ever homeless: 77.3% Mental health diagnosis: 86.4% Ever HCV positive: 33.3% | Reported of FCH provides a better high: 59% |
| Mazhnaya et al (2020)^41^ | Prefer drugs containing fentanyl (n=135) | Median (IQR): 35 (28-40) | Number of injections per day: 0: 5.4% 1-2: 10.8% 3-5: 51.5% 5+: 32.3% | Male: 48.9% Female: 51.1% | Injection drug use past 6 months Fentanyl: 83.7% Heroin: 97.8% Buprenorphine/suboxone: 25.2% Painkillers: 25.2% Crystal meth: 76.3% Speedball: 55.6% Cocaine: 49.6%  Other drugs use past 6 months: Smoked heroin: 23.7% Swallowed fentanyl: 17.8% Swallowed painkillers: 34.8% Buprenorphine/suboxone: 30.4% | Number of overdoses experienced in past 6 months 0 :41.5% 1-2: 29.6% 3-5: 15.6% 5+: 13.3% | White, non-Hispanic: 84.4% | High school graduate: 71.6% Single: 55.2% Sexual Minority: 17.2% Homeless: 60% Unemployed: 71.9% Food insecurity: 68.9% Transactional sex work in past 6 months: 68.9% Arrested in past 6 months: 37.8% | N/R |
| Mitra et al (2020)^49^ | Self-reported intentional fentanyl users (n=386) | Median (IQR): 39 (28.2-50.4) | Injection drug use: 87% | Male: 66.1% Female: 33.9% | Heroin use: 60.9% Prescription opioid use: 3.6% Stimulant use: 43.5% Opioid agonist treatment: 58.8% | High/moderate perceived risk of fentanyl overdose (men; women): 50.4%; 40.5% Low/no perceived risk of fentanyl overdose (men; women): 49.6%; 59.5% | Non-white: 47.5% White: 52.5% | Downtown east side residency: 63.0% Incarceration: 9.9% Exchanged money for sex: 11.4% Experienced violence: 13.6% | N/A |
| Morales et al (2019)^42^ | Preference for fentanyl (n=83) | Median (IQR): 38 (32-46) | Injection drug use: 83.1% | Male: 62.7%  Female: 37.3% | Prescribed opioid use: 53% Medication-assisted treatment: 75% Nonprescription therapeutic opioid use: 50.6% Nonprescription use of tranquilizers or benzodiazepine medication: 56.6% Daily illicit drug use: 91.6% Heroin injection: 78.3% Heroin, smoked or snorted: 32.5%  Crack cocaine use: 73.5% Snort powdered cocaine: 19.3% Cocaine injection: 44.6% Speedball injection: 53% | Never: 25.3% More than a year ago: 31.3% Within the last year: 43.4% Suspected due to fentanyl: 86.1% | White: 59% Black: 24.1% Hispanic: 7.2% Other/multiracial: 9.6% | Currently homeless: 69.9% Main sources of income last 3 months illegal work: 63.9% Arrested / incarcerated, last year: 59.0% | N/R |

*Significant finding that this subgroup would more likely take fentanyl intentionally

**Self-reported heroin use; UDS came back positive for fentanyl

*Abbreviations: N/R: not reported, N/A: not applicable, FCH: fentanyl-contaminated heroin*

**Table S4. Non-intentional fentanyl using subpopulations among studies comparing intentional fentanyl with non-intentional fentanyl (N=9)**

|  | **Comparison group** | **Age** | **IV use** | **Gender** | **Substance use patterns** | **Overdose** | **Racial/Ethnic** | **Socioeconomic** | **Motivation of use** |
| --- | --- | --- | --- | --- | --- | --- | --- | --- | --- |
| Antoine et al (2021)^37^ | Non-intentional fentanyl use** (n=1) | 26‐40: 100% (1/1) | 3 years | Female: 100% (1/1) | Heroin/fentanyl use intravenous/intranasal: 100% (1/1) | N/R | Non-Caucasian: 100% (1/1) | N/R | N/R |
| Chandra et al (2021)^38^ | No purposeful fentanyl use (n=59) | Mean (SD): 43.1 (9.3) | Inject daily: 18.3% | Male: 49.2% Female: 50.9% | Heroin: 15.3% Cocaine: 78.9% Poly drug 85.6% | Non-fatal OD in past year: 11.9% | White: 74.6% Non-white: 25.4% | High school graduate: 67.8% Income level <$10,000: 78.0% Currently married/living with partner: 23.7% Homeless in past year: 47.5% | N/R |
| Geddes et al (2018)^52^ | No recent fentanyl injection (n=655) | <30 years: 12% 30–39 years: 37% 40–49 years: 32% >49 years: 19% | Injecting initiation (years) <9: 14% 10–19: 32% 20–29: 32% >29: 18%  Daily injection: 61% Public injecting: 40% Reused syringes: 23% Used another’s used drug preparation item: 36% Used another’s used syringe: 23%  All last month | Female: 30% Male: 69%  Heterosexual: 80% Bisexual: 10% Homosexual: 3% | Currently in OST: 34%  Not currently in OST: 62% | Overdose in last 12 months: 21% | Indigenous Australian: 14% non-indigenous: 84% | HCV negative: 38% HCV positive: 62% | N/A |
| Karamouzian et al (2020)^48^ | No fentanyl use (n=120) | ≥50: 66.6% 40–49: 33.3% 30–39: 26.1% 19–29: 36.6% | Preferred route of administration  injection: 16.8% non-injection: 54.6% | Men: 42.3% Women: 36% LGBTQ: 37.5% | Cannabis: 47.7%  Methadone: 20.5% Heroin/morphine: 9.0%  Oxycodone use: 14.2% Crystal meth: 24.4% Cocaine: 28.8% Crack: 35.3% Benzodiazepine: 34.2%  Polydrug: 13.1% | Experienced non-fatal overdose in the last 6 months: 22.2% | N/R | Unstable housing (current): 31.4% Paid employment: 56.3% Medium/large urban cities: 25.6% Small urban/rural communities: 63.39% | N/R |
| Kline et al (2021)^39^ | No lifetime overdoses (n=238) | Mean (SD): 41.02 (11.72) | IV Injection: 28.2% | Male: 48.3% | Often/always mixes opioids with one or more other drugs: 51.3% Heavy alcohol use: 17.2% | Mean number of lifetime overdoses: N/A | White: 46.4% African American: 36.1% Hispanic: 17.6% | Homelessness: 10.1% | Believes fentanyl is an important cause of overdose: 79.0% Believes fentanyl is a dangerous drug: 78.6% Prefers fentanyl to other opioids: 24.7% |
| Macmadu et al (2017)^40^ | No fentanyl-contaminated exposure use in prior 6 months (n=177) | Mean (IQR): 24 (22-27) | Injection drug use: 9.0% | Male: 64.4% Female: 36.6% | Heroin: 18.6% NMPO: 45.2% Cocaine: 7.9% Non-medical benzodiazepine: 26.6% Diverted pharmaceutical fentanyl: 6.8% | Ever experienced a non-fatal overdose: 22.0% | White, non-Hispanic: 57.1% Other: 42.9% | Education beyond high school: 50.9% Ever detained in jail: 45.8% Ever homeless: 51.4% Mental health diagnosis: 71.2% Ever HCV positive: 8.3% | N/R |
| Mazhnaya et al (2020)^41^ | Do not prefer drugs containing fentanyl (n=176) | Median (IQR): 37 (31-42) | Number of injections per day 0: 10.3% 1-2: 19.4 3-5: 42.9% 5+: 27.4% | Male: 67.1% Female: 32.9% | Injection drug use, past 6 months: Fentanyl: 57.4% Heroin: 86.9% Buprenorphine/suboxone: 29.6% Painkillers: 22.2% Crystal meth: 74.3% Speedball: 35.2% Cocaine: 30.7%  Other drug use past 6 months: Smoked heroin: 13.1% Swallowed fentanyl: 5.7% Swallowed painkillers: 28.4% Swallowed buprenorphine/suboxone: 31.3% | Number of overdoses experienced in past 6 months 0: 53.4% 1-2: 25.6% 3-5: 12.5% 5+: 8.5% | White, non-Hispanic: 93.8% | High school graduate: 72.2% Single: 49.4% Sexual Minority: 13.1% Self-homeless: 56.3% Unemployed: 62.5% Food insecurity: 65.3% Transactional sex work in past 6 months: 11.4% Arrested in past 6 months: 35.8% | N/R |
| Mitra et al (2020)^49^ | Self-reported unintentional fentanyl exposure (n=192) | Median (IQR): 44.7 (34.6-53.4) | Injection drug use: 80.7% | Male: 54.2% Female: 45.8% | Heroin use: 43.2% Prescription opioid use: 5.7% Stimulant use: 41.7% OAT: 55.7% | High/moderate perceived risk of fentanyl overdose (men; women): 52.0%; 62.5% Low/no perceived risk of fentanyl overdose (men; women): 48.0%;36.4% | Non-white: 50.0% White: 50.0% | Downtown eastside residency: 73.0% Incarceration: 8.3% Exchanged money for sex: 12% Experienced violence: 14.6% | N/R |
| Morales et al (2019)^42^ | Does not prefer fentanyl (n=225) | Median (IQR): 45 (37-52) | Injection drug use: 68.4% | Male: 59.1% Female: 40.9% | Prescribed opioid use: 53.3% Medication-assisted treatment: 70% Nonprescription therapeutic opioid use: 49.3% Nonprescription use of tranquilizers or benzodiazepine medication: 41.3% Daily drug use: 75.6% Heroin injection: 64% Heroin, smoked or snorted: 51.1%  Crack cocaine use: 69.3% Snort powdered cocaine: 28% Cocaine injection: 35.1% Speedball injection: 94 41.8% | Never: 37.3% More than a year ago: 17.3% Within the last year: 45.3% Suspected due to fentanyl: 91 (89.2%) | White: 29.3% Black: 45.3% Hispanic: 14.2% Other/multiracial: 11.1% | Currently homeless: 68.0% Main sources of income, last 3 months illegal work: 41.3% Arrested / incarcerated, last year: 42.2% | N/R |

*Significant finding that this subgroup would more likely take fentanyl intentionally

**Self-reported heroin use; UDS came back positive for fentanyl

Abbreviations: N/R, not reported; N/A, not applicable, FCH, fentanyl-contaminated heroin

**Table S5: Study quality table (N=41)**

|  |  | **Modified Newcastle Ottawa Quality Assessment Scale for Case Control Studies** | | | | | | | | |
| --- | --- | --- | --- | --- | --- | --- | --- | --- | --- | --- |
|  |  | **SELECTION (max 4)** | | | | **COMPARABILITY (max 2)** | **EXPOSURE (max 3)** | | |  |
|  | **Methodology of study** | **Is the case definition adequate?** | **Representativeness of the cases** | **Selection of controls** | **Definition of controls** | **Comparability of cases and controls on the basis of the design or analysis** | **Ascertainment of exposure** | **Same method of ascertainment for cases and controls** | **Non-response rate** | **Score (/9)** |
| Uuskula et al (2017) | Cross-sectional & observational | + | + |  | + | + | + | + |  | 6 |

|  |  | **Modified Newcastle Ottawa Quality Assessment Scale for COHORT Studies** | | | | | | | | |
| --- | --- | --- | --- | --- | --- | --- | --- | --- | --- | --- |
|  |  | **SELECTION (max 4)** | | | | **COMPARABILITY (max 2)** | **OUTCOME (max 3)** | | |  |
|  | **Methodology of study** | **Representativeness of the exposed cohort** | **Selection of the non-exposed cohort** | **Ascertainment of exposure** | **Demonstration that outcome of interest was not present at the start of the study** | **Comparability of cohorts on the basis of the design or analysis** | **Assessment of outcome** | **Was follow-up long enough for outcomes to occur?** | **Adequacy of follow up of cohorts** | **Score (/9)** |
| Buresh et al (2019) | Cross-sectional study | + |  |  |  |  |  | + | + | 3 |

|  |  | **Modified Newcastle Ottawa Quality Assessment Scale for CROSS SECTIONAL Studies** | | | | | | | |
| --- | --- | --- | --- | --- | --- | --- | --- | --- | --- |
|  |  | **SELECTION (max 5)** | | | | **COMPARABILITY (max 2)** | **OUTCOME (max 3)** | |  |
|  | **Methodology of study** | **Representativeness of the sample** | **Sample size** | **Non-respondents** | **Ascertainment of the exposure** | **Comparability based on the study design or analysis** | **Assessment of the outcome** | **Statistical test** | **Score (/10)** |
| Amlani et al (2015) | Cross-sectional study | + |  | + | + | ++ | ++ | + | 8 |
| Bach et al (2020) | cross-sectional study | + |  | + | ++ | ++ | ++ | + | 9 |
| Balsamo et al (2021) | Semiautomatic information retrieval algorithm Reddit |  |  |  |  |  |  |  |  |
| Buresh et al (2019) | Cross-sectional study quantitative survey |  | + |  | ++ | ++ | ++ | + | 8 |
| Chandra et al (2021) | Cross-sectional | + | + |  | ++ | ++ | + | + | 8 |
| Geddes et al (2018) | Cross sectional | + | + | + | + | + | + | + | 7 |
| Gryczynski, et al (2019) | Review of treatment records & anonymous survey | + |  |  | + |  | ++ | + | 5 |
| Karamouzian et al (2020) | Cross-sectional | + | + | + | + | + | + | + | 7 |
| Kilwein et al (2018) | Descriptive study, fentanyl questionnaire | + | + |  | ++ |  | + |  | 5 |
| Kline et al (2021) | Cross-sectional | + | + |  | ++ | ++ | + | + | 8 |
| Krause et al (2017) | Cross sectional- Urine analysis & questionnaire |  |  |  | ++ |  | ++ | + | 5 |
| Macmadu et al (2017) | Cross-sectional | + | + |  | + |  | + | + | 5 |
| Mazhnaya et al (2020) | Cross-sectional | + | + |  | + | ++ | + | + | 7 |
| Mitra et al (2020) | Cross-sectional study with interviews & surveys. | + | + |  | + | ++ | + | + | 7 |
| Nolte et al (2020) | Interview & survey | + | + |  | ++ |  | ++ | + | 7 |
| O'Rourke et al (2019) | Cross sectional survey with statistical analysis | + | + |  | + |  | + | + | 5 |
| Park et al (2019) | Survey, interview | + | + |  | ++ |  | + | + | 6 |
| Uuskula et al (2017) | Cross-sectional & observational | + | + | + | ++ |  | ++ | + | 8 |
| Wallace et al (2019) | Cross-sectional survey | + | + |  | + |  | + | + | 5 |

|  |  | **CASP Tool for Qualitative Appraisal** | | | | | | | | | | | |
| --- | --- | --- | --- | --- | --- | --- | --- | --- | --- | --- | --- | --- | --- |
|  | **Methodology of study** | **Was there a clear statement of the aims of the research?** | **Is a qualitative methodology appropriate?** | **Was the research design appropriate to address the aims of the research?** | **Was the recruitment strategy appropriate to the aims of the research?** | **Was the data collected in a way that addressed the research issue?** | **Has the relationship between researcher and participants been adequately considered?** | **Have ethical issues been taken into consideration?** | **Was the data analysis sufficiently rigorous?** | **Is there a clear statement of findings?** | **How valuable is the research?** | **Score (/10)** |  |
| Bardwell et al (2021) | Qualitative semi-structured interviews | + | + | + | + | + |  | + | + | + | Very (+) | 9 |  |
| Ciccarone et al (2017) | Qualitative rapid study & semi-structured interview | + | + | + | + | + |  | + | + | + | Very (+) | 9 |  |
| Daniulaityte et al (2019) | Semi structured interviews & urine drug screen | + | + | + | + | + |  |  | + | + | Very (+) | 8 |  |
| Eiden et al (2017) | Case report |  |  |  |  |  |  |  |  |  |  |  |  |
| Firestone et al (2009) | Exploratory (qualitative study | + | + | + |  | + |  |  |  | + | Moderately valuable (+) | 6 |  |
| Gunn et al (2021) | Qualitative interviews | + | + | + | + | + |  |  | + | + | Very (+) | 8 |  |
| Kenney et al (2018) | Survey | + | + | + | + | + | + |  | + | + | Very (+) | 9 |  |
| McLean et al (2019) | Qualitative interview & surveys | + | + | + | + | + |  |  |  | + | Very (+) | 7 |  |
| Moeller et al (2020) | Thematic analysis of a public internet forum Flashback | + | + | + | + | + |  |  | + | + | Very (+) | 8 |  |
| Nolte et al (2020) | Interview & survey | + | + | + | + | + |  | + | + | + | Very (+) | 9 |  |
| Silverstein et al (2019) | Qualitative interviews | + | + | + |  | + |  |  | + | + | Very (+) | 7 |  |
| Weicker et al (2020) | Qualitative interviews | + | + | + | + | + |  |  |  | + | Very (+) | 7 |  |

|  |  | **Case Report Guidelines (CARE Checklist)** | | | | | | | | | | | | | |
| --- | --- | --- | --- | --- | --- | --- | --- | --- | --- | --- | --- | --- | --- | --- | --- |
|  | **Methodology of study** | **Title** | **Keywords** | **Abstract** | **Introduction** | **Patient information** | **Clinical findings** | **Timeline** | **Diagnostic assessment** | **Therapeutic intervention** | **Follow up & outcomes** | **Discussion** | **Patient perspective** | **Informed consent** | **Score (/13)** |
| Antoine et al (2021) | Case-series |  |  | + | + | + | + |  | + | + |  | + |  | + | 8 |
| Eiden et al (2017) | Case report |  |  |  |  | + | + | + | + | + |  |  |  |  | 5 |
| Gecici et al (2010) | Case report | + | + | + | + | + | + | + | + | + | + |  |  |  | 10 |
| Guerrieri et al (2017) | Case-series, Post-mortemand femoral blood analysis |  | + | + | + | + | + | + | + | + |  |  |  |  | 8 |
| Kimergard et al (2018) | Retrospective case review | + | + | + | + | + | + | + | + | + |  | + |  | + | 11 |
| Lyttle et al (2012) | Case report |  | + | + |  | + | + | + | + | + |  | + |  |  | 8 |
| Marquardt et al (2008) | Case report |  | + | + |  | + | + | + | + | + | + | + |  |  | 10 |
| Mrvos et al (2012) | Retrospective case review | + | + | + | + | + | + |  | + | + |  | + |  |  | 9 |
| Reeves et al (2002) | Case report |  |  |  |  | + | + | + | + | + |  |  |  |  | 5 |
| Tharp et al (2004) | Post-mortem analysis | + | + | + | + | + | + | + | + | + |  |  |  |  | 9 |
| Woodall et al (2008) | Post-mortem autopsy, blood analysis & toxicological findings | + | + | + | + | + | + | + | + | + |  |  |  |  | 9 |
